# Supporting the Conditions for Healthy Cognitive Aging in Place Through Integrated Care: A Retrospective Qualitative Case Study with a Longitudinal Documentary Component

**DOI:** 10.3390/bs16091621

**Published:** 2026-09-10

**Authors:** Libor Stráník, Iva Holmerová, Marie Dohnalová, Alžběta Bártová

**Affiliations:** 1 Centre of Expertise in Longevity and Long-Term Care (CELLO), Faculty of Humanities, Charles University, 18200 Prague, Czech Republic; iva.holmerova@gerontocentrum.cz (I.H.); alzbeta.bartova@fhs.cuni.cz (A.B.); 2 Department of Sociology, Faculty of Humanities, Charles University, 18200 Prague, Czech Republic; marie.dohnalova@fhs.cuni.cz

**Keywords:** aging in place, healthy cognitive aging, integrated care, cognitive load, context–mechanism–outcome configurations, social embeddedness, case study

## Abstract

Aging in place (AiP) and healthy cognitive aging (HCA) are closely interrelated concepts but seldom examined together within real-world care systems. Integrated care (IC) offers a framework to understand how community settings can support both. This qualitative case study examined a 25-year-old community-based integrated health and social care program in a rural Czech microregion. Utilizing a realist-informed context–mechanism–outcome framework, the study integrated data from documentary analysis, semistructured interviews, and participant observation. The findings suggest that the conditions facilitating AiP and HCA may be mutually supported through interrelated, context-dependent, cumulatively operated mechanisms, including environmental predictability, continuity of care, coordination across services, social embeddedness, supported autonomy, and reductions in cognitive and organizational burden and stress. IC is not a direct intervention, but acts as a context-dependent moderator that shapes the conditions for AiP and HCA. Behaviorally, these mechanisms minimize decision-making complexity and establish predictable interaction patterns that support everyday functioning. AiP and conditions relevant to HCA are mutually supported through shared underlying mechanisms embedded in care systems and community contexts. IC may facilitate this by fostering stable, person-centered environments that may reduce cognitive demands and support relational continuity. The study contributes to behavioral science by exploring how IC may shape conditions relevant to cognitive and functional outcomes in later life and provides actionable insights for the design of IC systems.

## 1. Introduction

Population aging is one of the most significant demographic transformations of the twenty-first century, reshaping health systems, social structures, and community life across the globe (United Nations, 2020). As longevity increases, a growing proportion of older adults live with chronic conditions, functional limitations, and varying degrees of cognitive change (Prince et al., 2015; World Health Organization, 2021). A substantial body of evidence indicates that most older adults prefer to age in place, seeking to maintain autonomy, activity, and independence for as long as possible while remaining embedded in familiar home and social environments (AARP, 2011; Rantz et al., 2005). This preference is not merely attitudinal but is reflected in actual living arrangements, as the majority of older people continue to reside within their communities rather than in institutional settings. Consequently, the extent to which this preference can be realised depends critically on the quality, stability, and accessibility of community-based support systems, which play a central role in shaping well-being and functional outcomes in later life (Wiles et al., 2012; Sixsmith & Sixsmith, 2008).

Cognitive health, in particular, plays a decisive role in determining whether older adults can maintain autonomy, meaningful social participation, and psychological well-being as they age (Depp & Jeste, 2006; World Health Organization, 2015; Beard et al., 2015). Understanding how community environments and care systems can support healthy cognitive aging has therefore become a priority for research, policy, and practice (Livingston et al., 2020).

Aging in place (AiP) has emerged as a key concept in this context. In gerontological research, it is commonly understood as the ability of older adults to live safely and independently in their own homes and communities while maintaining meaningful social roles and connections, regardless of age or functional ability (Wiles et al., 2012; Pani-Harreman et al., 2021), AiP reflects more than a preference for remaining at home. It encompasses a multidimensional set of conditions that enable older adults to sustain their identity, have access to the resources required to remain in their familiar environments, thereby supporting their independence, autonomy, and social connections (Wiles et al., 2012).

Research consistently shows that AiP is associated with higher life satisfaction, better psychological well-being, and stronger social integration (Vasunilashorn et al., 2012). From a psychological perspective, AiP supports continuity of self, familiar routines, and a sense of control—factors that shape conditions relevant to cognitive functioning and mental health (Rowles & Bernard, 2013). However, AiP is not guaranteed by physical residence alone. It depends on the interaction between individual capacities, environmental characteristics, social relationships, and the availability of supportive services (Lawton & Nahemow, 1973; Pani-Harreman et al., 2021). When these elements are misaligned, older adults may experience stress, disorientation, or social isolation, all of which can negatively affect cognitive health (Cacioppo & Hawkley, 2009).

Healthy cognitive aging (HCA) is a concept that focuses on the maintenance of cognitive functions—such as memory, attention, executive functioning, and orientation—that are essential for daily life and independent living (World Health Organization, 2015). Contemporary cognitive aging research emphasizes the heterogeneity of cognitive trajectories in later life (Cabeza et al., 2018; Nyberg et al., 2012). While some individuals experience marked decline, others maintain high levels of cognitive functioning well into advanced age. Theories such as cognitive reserve, brain maintenance, and the scaffolding theory of aging and cognition highlight the role of lifelong learning, physical activity, social engagement, and adaptive neural processes in shaping cognitive outcomes (Stern et al., 2019; Park & Reuter-Lorenz, 2009; Nyberg et al., 2012). Importantly, these theories also underscore the influence of environmental and social contexts (Livingston et al., 2020). Stable, predictable, and supportive environments can reduce cognitive and organisational burden, facilitate orientation, and promote engagement in cognitively stimulating activities (Evans, 2003; Fratiglioni et al., 2004). Conversely, fragmented care, unpredictable service environments, and social isolation can increase stress and reduce coping capacity—conditions associated in the literature with worse mental health and, in population studies, higher dementia risk (Cacioppo & Hawkley, 2009; Livingston et al., 2020). In our study, HCA is conceptualized not as a demonstrated clinical outcome, but as a theoretical and interpretive lens through which to examine the supportive capacity of care environments. Despite the conceptual overlap between AiP and HCA, research rarely examines them together. AiP studies often focus on housing, environmental adaptations, or social support, while HCA research tends to emphasize neurobiological and psychological determinants (Wiles et al., 2012; Stern et al., 2019). Yet the ability to age in place is deeply intertwined with cognitive functioning: cognitive health is associated with the ability of older adults to manage daily tasks, navigate their environment, and make decisions, while supportive environments and stable care systems help maintain conditions relevant to cognitive functioning by reducing stress and providing opportunities for engagement (Lawton & Nahemow, 1973; Livingston et al., 2020). The relationship between AiP and HCA is therefore reciprocal and dynamic, shaped by the broader sociotechnical systems in which older adults live (Sixsmith & Sixsmith, 2008).

Integrated care (IC) offers a promising framework for understanding how community settings can support both AiP and HCA. Integrated care refers to the coordinated, person-centered, and continuous provision of health and social services across providers, sectors, and settings (Kodner & Spreeuwenberg, 2002; Valentijn et al., 2013). It seeks to overcome the fragmentation that often characterizes care systems, where older adults must navigate complex and disconnected services (Goodwin et al., 2014). Fragmentation can create uncertainty and increase system complexity and user burden, potentially disrupting continuity and undermining psychological well-being—particularly for those navigating multiple providers and eligibility rules (Greer et al., 2019; Sørensen et al., 2012). In contrast, integrated care aims to create predictable, comprehensible, and stable care trajectories that align with individuals’ needs and preferences (Valentijn et al., 2013). From a psychological perspective, integrated care can reduce stress, enhance orientation, and support coping by providing consistent relationships with providers, clear communication, and coordinated transitions between services (Kodner & Spreeuwenberg, 2002). However, the capacity of integrated care to reduce such burden may be moderated by individual factors. Older adults do not enter these systems with uniform abilities; those with low health literacy or high nursing complexity remain particularly vulnerable to fragmentation and may benefit differently from integrated mechanisms (Cocchieri et al., 2025).

However, the potential of integrated care to support AiP and HCA remains insufficiently explored. Research on integrated care focuses on clinical outcomes, service efficiency, or organizational processes rather than on cognitive health or the lived experience of older adults in community settings (Goodwin et al., 2014). Even fewer studies examine long-term, real-world integrated care systems that have evolved over decades. Such systems offer unique opportunities to analyze how stable governance structures, interorganizational relationships, and community-based service networks shape the conditions for aging in place and cognitive well-being in community (Valentijn et al., 2013). Understanding these mechanisms is particularly important in rural regions, where demographic aging, limited service availability, and geographic dispersion pose additional challenges.

This study addresses these gaps by examining a 25-year integrated health and social care program operated by a voluntary association of municipalities in a rural microregion of the Czech Republic. The program represents a rare example of a long-standing, community-based integrated care system that has maintained its core principles for more than two decades. Its stability, sociotechnical infrastructure, and community governance provide a rich context for analyzing how integrated care can support aging in place and healthy cognitive aging. Unlike short-term projects or pilot interventions, this program allows for the examination of structural, organizational, and behavioral mechanisms that unfold over time.

Although our research does not directly investigate cognitive processes or psychological interventions, it provides an in-depth sociotechnical analysis of an integrated health and social care system in a specific rural microregion. Aging in place is inherently dependent on sociotechnical infrastructure and on the availability, coordination, and accessibility of services needed to support ageing populations (Sixsmith & Sixsmith, 2008). Our case study explores the ways in which organisational structures, inter-service relationships, and system-level interactions may facilitate or hinder the conditions conducive to AiP and cognitive well-being. This systems perspective contributes to understanding the structural and organisational prerequisites for effective community support of older adults’ healthy cognitive ageing in place.

To guide the analysis, we use a realist-informed logic of context–mechanism–outcome (CMO) patterns (Pawson & Tilley, 1997). This approach does not constitute a full realist evaluation; rather, it provides a structured way to identify how specific contexts trigger mechanisms that produce outcomes relevant to AiP and HCA.

The study addresses two study aims:

(1) Identify the theoretical and behavioral interrelationships between the concepts of aging in place and healthy cognitive ageing as a lens for analyzing real-world health and social care delivery.
(2) Analyse how integrated care may relate to conditions plausibly relevant to both concepts, and explore its potential moderating role in their interconnection. From a behavioral perspective, these system-level characteristics can be understood as shaping how individuals perceive, interpret, and respond to their care environment, particularly by influencing decision-making demands, perceived predictability, and coping processes in everyday life.

Our research question is as follows:

To what extent, in what way, and within what limits can conditions relevant to aging in place and to healthy cognitive aging be supported in a given region through integrated health and social care?

This article presents findings from a part of a case study conducted by the Centre of Expertise in Longevity and Long-term Care at the Faculty of Humanities, Charles University, Prague. This case study is part of a broader research project exploring the integration of health and social care in long-term care for older adults in the Czech Republic. The project is being carried out as part of the principal author’s PhD dissertation, and this article serves as a contribution to it.

## 2. Theoretical Background

Aging is increasingly conceptualized as a dynamic, multidimensional process shaped by the interplay of biological, psychological, social, and environmental factors (Rowe & Kahn, 1997; World Health Organization, 2015). Within this landscape, three concepts—Aging in Place (AiP), Healthy Cognitive Aging (HCA), and Integrated Care (IC)—have become important reference points in contemporary gerontology, health psychology, and long-term care policy (Sixsmith & Sixsmith, 2008; Valentijn et al., 2013). Although these concepts are often examined separately, they are deeply interconnected. AiP provides the environmental and social context in which cognitive aging unfolds (Wiles et al., 2012); HCA captures the neurocognitive and psychological processes that enable autonomy and participation (Cabeza et al., 2018; Stern et al., 2019); and IC represents the organizational and systemic structures that can support both through coordinated service delivery and continuity of care (Kodner & Spreeuwenberg, 2002; Valentijn et al., 2013).

This section synthesizes the theoretical foundations of these concepts and outlines the analytical lens used in this study.

### 2.1. Aging in Place (AiP)

Aging in place is commonly conceptualised as the ability to live safely and independently in one’s own home and community while sustaining identity, autonomy, and social connectedness, even as functional capacity changes (Wiles et al., 2012; Pani-Harreman et al., 2021). While early formulations emphasized remaining in one’s dwelling, contemporary research highlights AiP as a multidimensional and relational process (Wiles et al., 2012). Wiles et al. (2012) argue that AiP encompasses emotional attachment, identity, autonomy, and social connectedness, reflecting the complex ways in which older adults experience “place.”

#### 2.1.1. AiP as a Dynamic Process

AiP is not a static condition but a process shaped by the interaction between individual capacities, environmental characteristics, and available support systems (Lawton & Nahemow, 1973; Pani-Harreman et al., 2021). Older adults value the continuity of familiar routines, the symbolic meaning of home, and the social ties embedded in their neighborhoods. These elements contribute to psychological well-being and shape conditions relevant to cognitive functioning by providing stability, predictability, and a sense of control (Rowles & Bernard, 2013; Wiles et al., 2012). While AiP is broadly linked to life satisfaction, our study specifically focuses on how environmental stability may support cognitive-specific pathways—such as reducing executive load and preserving orientation—distinguishing these from general psychological well-being or quality of life.

#### 2.1.2. Environmental Gerontology and Person–Environment Fit

The ecological model of aging (Lawton & Nahemow, 1973) posits that well-being in later life depends on the balance between individual competence and environmental press. When environments compensate for declining competence—through accessibility, supportive services, or social resources—older adults can maintain autonomy and functional independence. Conversely, environments that are overly demanding or insufficiently supportive can increase stress, reduce coping capacity, and undermine cognitive functioning (Pani-Harreman et al., 2021).

In behavioural terms, such stability may allow individuals to rely on established routines and reduce the need for ongoing adaptation and problem-solving in daily life.

A complementary way to conceptualise AiP is through the capability approach, which emphasises that well-being depends on real opportunities to achieve valued ways of living rather than on preferences alone. From this perspective, ageing in place is not simply a choice to remain at home but a capability that is expanded or constrained by housing quality, service availability, transport, social relations, and local governance. This lens is particularly relevant in rural settings, where structural constraints can limit the practical feasibility of AiP even when the preference is strong (Sen, 1999; Nussbaum, 2000).

It is essential to acknowledge that the ‘environmental press’ is not experienced uniformly. Individual capacities to navigate these environments are significantly moderated by health literacy and the complexity of nursing needs; those with lower literacy levels are more susceptible to system fragmentation and may require more intensive ‘scaffolding’ from the integrated care system to maintain autonomy (Cocchieri et al., 2025).

#### 2.1.3. Social Embeddedness and Community Resources

AiP is also shaped by social capital and community embeddedness. Neighborhood ties, informal support networks, and opportunities for social participation play significant roles in maintaining psychological well-being and cognitive engagement (Sixsmith & Sixsmith, 2008). Social isolation, by contrast, is associated with cognitive decline, depression, and reduced quality of life (Cacioppo & Hawkley, 2009; Livingston et al., 2020). Older adults who remain socially connected and engaged in meaningful activities are more likely to maintain cognitive functioning and emotional resilience (Livingston et al., 2020).

#### 2.1.4. Cultural and Contextual Variations

AiP is not experienced uniformly across contexts and cultures. Research shows that cultural norms, family structures, and welfare systems influence how older adults conceptualize “place” and independence (Sixsmith & Sixsmith, 2008; Wiles et al., 2012). These variations underscore the need for flexible, context-sensitive approaches to supporting AiP.

#### 2.1.5. AiP as a Determinant of Cognitive Health

From a psychological perspective, AiP may create conditions consistent with supporting cognitive functioning by providing environmental stability, familiar routines, and opportunities for engagement (Rowles & Bernard, 2013). Stable environments reduce cognitive and organisational burden by minimizing uncertainty and limiting the need for continuous reorientation, while predictable routines support orientation and memory (Evans, 2003; Fratiglioni et al., 2004). Conversely, disruptions in living arrangements, service fragmentation, or environmental stressors can negatively affect conditions relevant to cognitive health (Evans, 2003; Cacioppo & Hawkley, 2009). AiP can provide a crucial foundation by shaping conditions relevant to healthy cognitive aging.

### 2.2. Healthy Cognitive Aging (HCA)

Healthy cognitive aging (HCA) refers to the maintenance of cognitive functions—such as memory, attention, executive functioning, and processing speed—that enable older adults to navigate daily life, make decisions, and participate in social and community activities (World Health Organization, 2015). HCA is not defined by the absence of cognitive change but by the preservation of functional abilities that support autonomy and well-being (Depp & Jeste, 2006).

From the range of models and theories related to cognitive aging, we focus on three key frameworks relevant to this study: cognitive reserve, brain maintenance, and the scaffolding theory of aging and cognition.

In the context of this our qualitative inquiry, HCA is utilized as an interpretive framework to examine the maintenance of cognitive functions—such as memory, attention, and executive functioning—that support autonomy and community participation. Rather than a clinically measured outcome, HCA serves here as a sensitizing concept to identify environmental conditions that may be conducive to cognitive well-being.

#### 2.2.1. Heterogeneity of Cognitive Trajectories

Cognitive aging is characterized by substantial individual variability. Some older adults experience minimal decline, while others show accelerated deterioration (Cabeza et al., 2018; Nyberg et al., 2012). This variability reflects the interaction of neurobiological processes, lifestyle factors, social environments, and structural conditions (Prince et al., 2015; Livingston et al., 2020).

#### 2.2.2. Time and Variability

Cognitive aging is a dynamic process characterized by both short-term fluctuations and long-term trajectories that are sensitive to changes in environment, social interactions, and daily routines (Evans, 2003; Fratiglioni et al., 2004). This perspective aligns closely with AiP, which emphasizes continuity and stability.

#### 2.2.3. Cognitive Reserve

Cognitive reserve explains why individuals with similar levels of neuropathology may exhibit different cognitive outcomes (Stern et al., 2019). Built through lifelong cognitive stimulation, social engagement, and education, cognitive reserve enables more efficient or flexible cognitive processing.

#### 2.2.4. Brain Maintenance

Brain maintenance emphasizes the preservation of neural integrity through healthy lifestyle behaviors, including physical activity, sleep, and vascular health (Nyberg et al., 2012). Individuals with better brain maintenance tend to experience slower cognitive decline.

#### 2.2.5. Scaffolding Theory of Aging and Cognition (STAC)

The scaffolding theory (STAC) (Park & Reuter-Lorenz, 2009) proposes that the aging brain adapts to structural decline by recruiting alternative neural networks. These compensatory processes help maintain cognitive performance and are influenced by environmental and behavioural factors.

#### 2.2.6. Environmental and Social Determinants of HCA

Environmental stability, social relationships, and structural conditions play a crucial role in shaping cognitive trajectories (Livingston et al., 2020). Stress, uncertainty, and fragmented care can increase cognitive load and reduce coping capacity, potentially accelerating decline (Cacioppo & Hawkley, 2009). Conversely, predictable environments and supportive relationships can promote cognitive engagement.

#### 2.2.7. Operationalizing HCA

In this study, Healthy Cognitive Aging is utilized as a theoretical lens to examine the conditions supporting the capacity to maintain cognitive well-being rather than a demonstrated neurocognitive outcome. HCA is conceptualized as a set of conditions relevant to cognitive well-being—such as sustained orientation, social engagement and reduced navigational stress. The study examines the care system’s capacity to provide ‘cognitive scaffolding’ (STAC) without direct longitudinal psychometric measures. Environmental influences can also be understood behaviourally, as they shape the demands placed on attention, decision-making, and adaptive responses in everyday situations and responses to changing care situations.

### 2.3. Integrated Care (IC)

Integrated care has become an increasingly important concept in health and social care policy, reflecting the recognition that older adults’ needs span multiple sectors (Kodner & Spreeuwenberg, 2002; Valentijn et al., 2013). Integrated care refers to the coordinated, person-centered, and continuous provision of services across providers, sectors, and settings.

#### 2.3.1. Fragmentation as a Structural Problem

Health and social care systems have historically developed in parallel, resulting in fragmentation and coordination challenges (Goodwin et al., 2014). This fragmentation produces gaps in continuity, duplication of services, and unpredictable care transitions. For older adults, navigating such systems can be cognitively demanding and stressful, particularly when cognitive capacity is reduced (Greer et al., 2019).

From a behavioral standpoint, fragmentation can be interpreted as increasing the number of required decisions, interactions, and adjustments, thereby raising cognitive and emotional demands on users.

#### 2.3.2. Levels of Integration

Valentijn et al. (2013) distinguish three levels of integration: micro (individual care), meso (organizational), and macro (policy). Functional and normative integration—shared values, trust, and communication—are essential for sustaining these structures.

#### 2.3.3. Continuity of Care as Psychological Stability

Continuity, together with coordination and person-centered care, is a core component of integrated care (Kodner & Spreeuwenberg, 2002). It reduces uncertainty, supports orientation, and enhances coping by providing a stable and comprehensible care environment (Greer et al., 2019).

Conversely, continuity may enable individuals to anticipate interactions and rely on familiar patterns of behaviour, reducing the need for continuous reorientation.

#### 2.3.4. Integrated Care as a Community-Level Intervention

Integrated care can be understood not only as an organizational model but also as a community-level intervention shaping the environments in which older adults live (Goodwin et al., 2014). By creating stable and accessible service ecosystems, integrated care is conceptualized as a conditional moderator. It may support the synergy between AiP and HCA by structurally decompressing the individual’s environment and reducing the cognitive and emotional burden of care navigation.

### 2.4. Interdisciplinary Analytical Framework: Realist-Informed CMO Configurations

This study uses a realist-informed logic of context–mechanism–outcome (CMO) configurations as an analytical framework for understanding how integrated care supports AiP and HCA (Pawson & Tilley, 1997). Rather than conducting a full realist evaluation, the CMO framework is used to structure explanations of how contexts activate mechanisms leading to specific outcomes.

Contexts include environmental stability, governance structures, service availability, and community characteristics. Mechanisms refer to psychological processes such as predictability, reduced cognitive load, orientation, coping, and social anchoring. Outcomes include the ability to age in place, maintain cognitive functioning, and sustain autonomy.

In line with realist-informed reasoning, we treat mechanisms not as internal cognitive events that can be directly observed, but as socially and organisationally moderated processes that explain how participants respond to particular system conditions. In this study, for example, continuity is operationalised as sustained relational contact with familiar providers over time; coordination as the practical alignment of tasks, information, and responsibilities across organisations; and social embeddedness as stable, repeated interactions within local networks that provide both practical assistance and emotional anchoring. These operationalisations facilitate a transparent, qualitative interpretation of how specific contexts may trigger mechanisms relevant to cognitive well-being. In line with realist principles, these mechanisms are treated as theoretical inferences regarding how participants respond to system conditions, rather than as directly observed internal neurocognitive events. This interdisciplinary approach integrates insights from environmental gerontology, cognitive aging research, and health psychology, clarifying how system-level arrangements shape individual cognitive and functional outcomes.

## 3. Materials and Methods

### 3.1. Study Design

This study adopts a retrospective qualitative case study design with a longitudinal documentary component to examine how a long-term, community-based integrated health and social care program may relate to conditions supporting Aging in Place (AiP) and healthy cognitive aging (HCA) in a rural microregion of the Czech Republic. The documentary component covers a twenty-five-year period, from 2000 to 2024, whereas interviews and observations were conducted in 2025 and provide both a contemporary and a retrospective perspective on the functioning of the integrated health-and-social care system in the region (the Programme). Where relevant, these two evidentiary strands are analytically distinguished in the presentation of findings (Section 4).

The retrospective qualitative case study design with a longitudinal documentary component allows for the examination of how a long-term, community-based integrated health and social care program can support Aging in Place (AiP) and Healthy Cognitive Aging (HCA) in a rural microregion of the Czech Republic, specifically over a period of twenty-five years, from 2000 to 2024 while utilizing Healthy Cognitive Aging (HCA) as a theoretical and interpretive lens rather than a clinical outcome measures.

A case study approach was chosen because the program represents a complex sociotechnical system whose functioning is deeply embedded in historical development, institutional arrangements, and everyday practices. Case studies are particularly well suited for analysing such context-dependent phenomena, enabling the tracing of causal pathways, the identification of patterns, and the examination of how specific contextual conditions shape outcomes over time (Yin, 2018; Stake, 1995).

The program under study (the Programme) has been implemented continuously for more than 25 years and comprises 43 sequentially developed projects. Crucially, the Programme was not designed to promote AiP or HCA. Its purpose was to provide integrated health and social care in a geographically defined region. AiP and HCA therefore constitute a parallel analytical perspective applied to an existing system, rather than the Programme’s original design logic. This required an analytical strategy capable of orienting the research within a complex empirical landscape and of identifying how integrated care arrangements may create or inhibit conditions relevant to AiP and HCA.

To structure this explanatory work, we employed context–mechanism–outcome (CMO) configurations. While CMO configurations are typically used within realist evaluation to develop middle-range theory (Pawson & Tilley, 1997), in this study they are applied as a heuristic analytical tool to support systematic interpretation of qualitative data and enhance the communicability and transferability of findings.

### 3.2. Setting

The research was conducted in a rural microregion composed of 27 municipalities that voluntarily formed an association to coordinate long-term care services. The region is characterised by demographic ageing, limited public transportation, uneven access to specialised health services, and the outmigration of younger residents—conditions typical of rural Central Europe. Over the past quarter century, the municipalities jointly developed a coordinated system of residential long-term care, home care, social services, volunteer programs, community nursing, and municipal support structures. This system evolved incrementally, guided by shared governance, stable leadership, and long-term collaboration among local actors.

The demographic profile of the region—marked by high proportions of older adults and increasing prevalence of chronic conditions—creates strong pressures on local care systems. These characteristics make the microregion an informative setting for examining how integrated care can support AiP and HCA under resource-constrained conditions and how community-based systems adapt to demographic and institutional change.

### 3.3. Analytical Workflow

**Figure 1:**
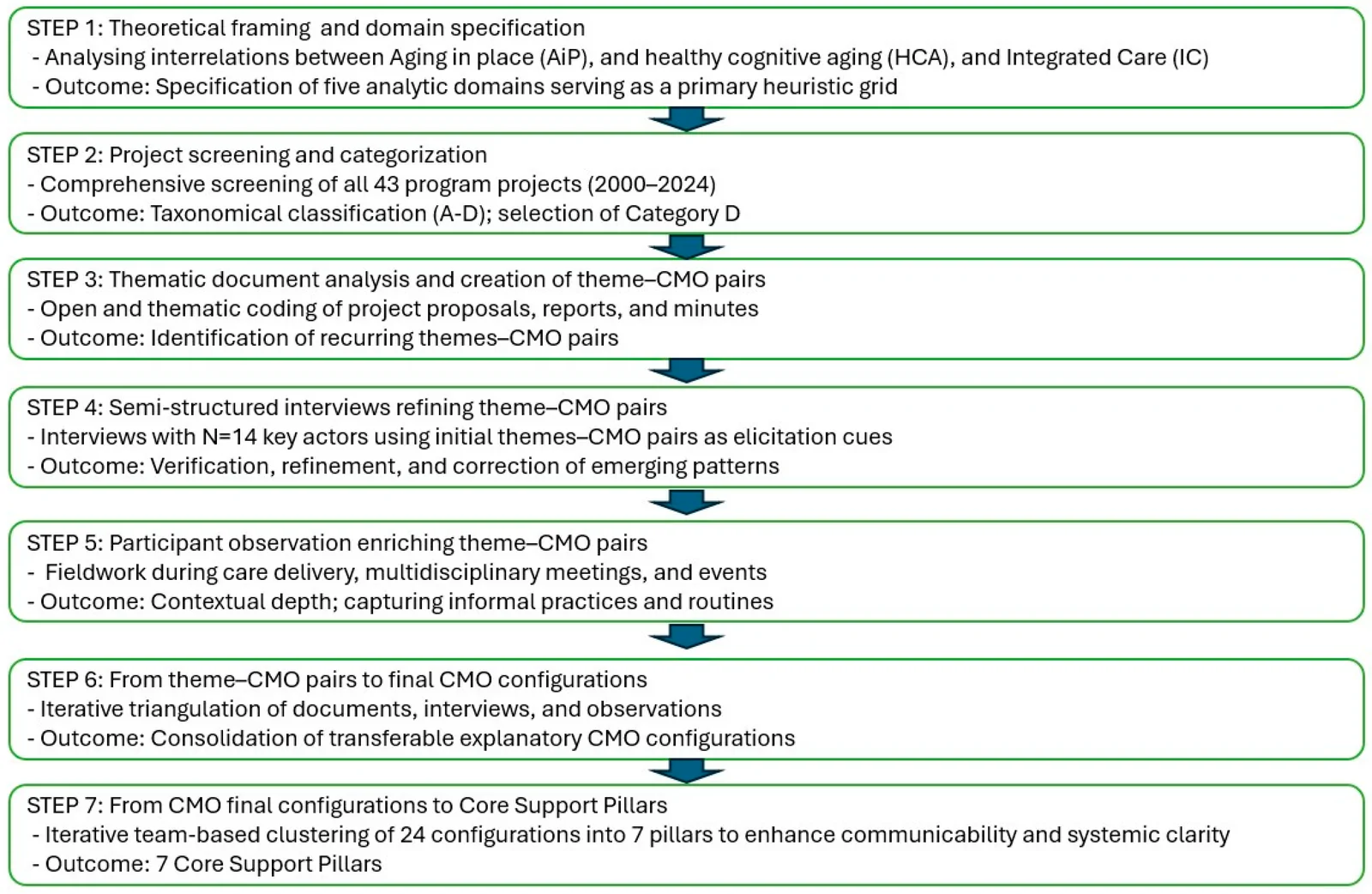
The Seven-Step Analytical Workflow of the Study. This schema delineates the iterative research process moving from initial theoretical framing and domain specification, through project screening and taxonomic classification, to qualitative stakeholder triangulation via documents, semi-structured interviews, and participant observations, culminating in the formulation of transferable Context–Mechanism–Outcome (CMO) configurations and in the synthesis of final CMO configurations into Core Support Pillars.

The study followed a six-step analytical workflow, each step building on the previous one to progressively refine the understanding of how integrated care shapes conditions relevant to AiP and HCA. The workflow is summarised in Figure 1.

*Step 1: Theoretical framing and domain specification*

We conducted a theoretical examination of the interrelationships among AiP, HCA, and IC, drawing on interdisciplinary literature in gerontology, cognitive aging, and health systems research (Valentijn et al., 2013; Stern et al., 2019). This analysis produced five analytic domains that capture the key conditions relevant to both AiP and HCA. These domains served as a primary heuristic grid to grasp the conceptual intersections between HCA and AiP, providing an initial organizational structure for orienting within the corpus of study materials and facilitating the operationalization of the research.

*Step 2: Project screening and categorization*

All 43 projects implemented within the Programme between 2000 and 2024 were assessed for their relevance to AiP and HCA within these analytic domains (step 1). Each project was independently assessed by two researchers (L.S., A.B.) based on the analytical areas. In order to increase credibility and minimize subjectivity, any discrepancies in the classification into categories (A–D) were resolved through repeated mutual consultations with the participation of all team members until consensus was reached. Only the projects with the highest relevance (category D) were selected for in-depth analysis consistent with purposive qualitative sampling strategies (Patton, 2015). Programme projects and their relevance categorisation (A–D), including the Category D subset selected for in-depth analysis, are summarised in Section 4 (see below), the criteria for categorizing projects (A–D) are listed there too.

*Step 3: Thematic document analysis and creation of theme–CMO pairs*

For selected projects (Category D), we conducted a thematic analysis of documentation materials, including project documentation, internal correspondence, evaluation reports, and other supporting documents. The analysis was conducted using an inductive approach (Braun & Clarke, 2006), whereby we identified recurring themes within individual areas and linked them to corresponding CMO configurations. This resulted in theme–CMO pairs, which were used in subsequent steps of the workflow.

*Step 4: Semi-structured interviews refining theme–CMO pairs*

The theme–CMO pairs were used as prompts in semi-structured interviews with key stakeholders. This approach enabled the exploration of participant perspectives and the refinement of emerging explanations, consistent with established qualitative interviewing practices (Kvale & Brinkmann, 2009).

*Step 5: Participant observation enriching theme–CMO pairs*

Targeted participant observations were conducted to examine how configurations were enacted in practice. Observational methods allowed for the analysis of situated interactions and organisational routines, providing contextual depth (Spradley, 1980).

*Step 6: From theme–CMO pairs to final CMO configurations*

After repeated refinement across data sources, the theme–CMO pairs yielded the final CMO configurations. During this stage, themes functioned as temporary ‘carriers’ for the emerging CMO configurations; once the configurations were validated and refined through data triangulation, the themes were withdrawn, leaving the finalized CMO configurations as the primary analytical output. These configurations represent transferable explanatory patterns rather than context-specific descriptions, which is consistent with analytical approaches grounded in realism (Pawson & Tilley, 1997). The final CMO configurations for each domain identified in step 1 of the workflow are detailed in Section 4 (see below).

However, as the research progressed, it became evident that the initial five-domain grid, while useful for operationalization, did not fully capture the systemic synergy of the findings. Consequently, to enhance the communicability and clarity of our conclusions, the final CMO configurations were further synthesized into a more integrated structure of seven support pillars.

*Step 7: From final CMO configurations to Core Support Pillars*

In this final stage, the research team transitioned from the initial organizational grid (domains) to a more integrated and communicable explanatory structure. The final CMO configurations were grouped into support pillars.

To translate the rich empirical observations from the field into a cohesive framework, the final CMO configurations (n = 24, see Section 4) were interpreted through a narrative, four-dimensional qualitative lens. This process allowed us to distinguish between core systemic moderators—elements that structurally reshape the senior’s daily living conditions—and operational enablers, which represent necessary but localized technical and administrative routines.

Rather than applying rigid formal criteria and metrics, this final synthesis and interpretation focused on four descriptive characteristics of the care environment:

- Structural Scope: This perspective explores whether a pattern operates as a broader modifier of the social and physical environment, or functions as a localized, standalone service.
- Behavioral Scaffolding: Here, the focus is on how the care ecosystem actively eases executive cognitive load and stabilizes daily routines, as opposed to offering transactional support.
- Temporal Continuity: This dimension highlights patterns that rely on long-term environmental stability and accumulated systemic trust, rather than brief, episodic responses.
- Functional Synergy: This lens observes whether an interaction pattern simultaneously supports both independent living (Aging in Place) and conditions relevant to cognitive well-being (Healthy Cognitive Aging) through shared pathways.

Through this thematic integration, patterns embedded within the structure of daily life were synthesized into core support pillars (n = 7, see Results). The remaining operational patterns, while essential for daily care delivery (such as, e.g., technical case management or equipment lending), were conceptually integrated within these broader pillars rather than presented as standalone conclusions.

### 3.4. Case and Project Selection

#### 3.4.1. Case Selection

The case was selected using criteria typical for theoretically informed qualitative inquiry, including long-term existence, organisational stability, provision of integrated services, and embeddedness in community networks (Yin, 2018; Stake, 1995).

#### 3.4.2. Project Selection

The study examines a purposive sample of integrated care projects implemented between 2000 and 2024. All projects were screened for relevance using the five analytic domains. Only those assigned to category D were selected for in-depth analysis, ensuring focus on information-rich cases (Patton, 2015). While Category D projects were selected for in-depth analysis due to their direct impact on daily behavioral routines, Categories A–C were conceptualized as the necessary systemic and clinical foundation. These categories provide the macro-level stability and acute medical safety net within which the micro-level mechanisms of Category D can be activated.

### 3.5. Data Sources

An overview of the study data sources and their role in the CMO heuristic is provided in Table 1.

#### 3.5.1. Documentary Materials

Documentary analysis followed established qualitative approaches to analysing organisational records and institutional documents (Bowen, 2009). The document corpus comprised approximately 120 core documents originating from all 43 projects, and roughly 300 documents associated with the 25 projects classified as category D, including project proposals, project documentation, internal correspondence, evaluation reports, operational guidelines, and meeting minutes. For analytical purposes, documents were retained only if they directly related to activities within the Programme; purely administrative or duplicate records were excluded. These materials informed both the thematic analysis and the development of the initial CMO configurations, alternatively theme–CMO pairs.

#### 3.5.2. Semi-Structured Interviews

Semi-structured interviews were conducted using flexible protocols designed to elicit rich, contextualised accounts (Kvale & Brinkmann, 2009). Interviews were audio-recorded and transcribed verbatim.

To empirically refine and validate the initial CMO configurations derived from the documentary analysis, a structurally stratified sample of qualitative interviews was executed. The sample consisted of 14 participants representing key stakeholder groups. The sample of stakeholders was proportionally distributed across four stakeholder tiers to balance structural, operational, and experiential knowledge: macro-context architects (n = 2; executive and municipal leaders), meso-integration coordinators (n = 4; 1 case manager, 2 senior social workers, 1 head nurse),micro-mechanism activators (n = 4; 2 field-based professional caregivers and 2 home-care nurses), and outcome beneficiaries (n = 4; 2 informal family caregivers and 2 older adults with mild cognitive decline). The term ‘mild cognitive decline’ in this sample refers to participants with functional cognitive symptoms as identified by case managers for service eligibility, rather than a formal clinical diagnosis (e.g., MMSE/MoCA). This proportional distribution shifts the analytical weight toward front-line care delivery and lived experience, capturing the latent behavioral mechanisms that documents may omit. Data saturation was approached through iterative data collection and analysis, with thematic repetition observed across interviews (Guest et al., 2006). Interviews were conducted between February and June 2025 and lasted approximately 90 min. When analyzing interview data, a clear distinction was maintained between contemporaneous evidence derived from 2000–2024 documents and retrospective accounts provided by stakeholders in 2025, accounting for potential recall bias and the tendency to rationalize past decisions through current professional standards.

#### 3.5.3. Participant Observation

Participant observation was conducted to capture situated interactions, implicit behavioral routines, and the real-time activation of CMO mechanisms within their natural social context (Spradley, 1980). The observations were executed throughout 2025 (lasted approximately 60 min each) and systematically stratified across three distinct operational settings to mirror the program’s multi-level architecture. Specifically, the sample was proportionally allocated among care visits (n = 6), divided between direct home-care deliveries and decentralized Local Care Cottages to observe client–caregiver micro-interactions and the scaffolding of procedural memory, multidisciplinary team meetings (n = 3), encompassing cross-sectoral case management conferences to evaluate health-social integration protocols, and localized community events (n = 2), including village social gatherings to assess informal neighborly support networks. Field notes were systematically recorded in situ, focusing on tacit professional practices and behavioural adjustments that stakeholders often omit in verbal interviews, with all data fully anonymised and securely stored in compliance with data protection standards.

### 3.6. Analytical Approach

#### 3.6.1. Thematic Analysis

We conducted an inductive thematic analysis following the six-phase approach outlined by Braun and Clarke (2006). Data coding was performed using ATLAS.ti (version 24) and refined iteratively. The coding process involved a team of three analysts to ensure multi-perspectivity. Primary coding was performed by the lead researcher and one additional team member, followed by a secondary blind coding of a subset of the data (25%) by the third team member. Primary coding responsibilities were divided between the lead researcher (L.S.) and one team member (A.B.), with each analyst coding distinct portions of the dataset. To ensure inter-coder reliability, a 25% subset of the total material—comprising a randomly selected sample from each of the three data sources (documents, interview transcripts, and observation notes)—was subjected to secondary blind coding by the third analyst (I.H.). Any disagreements regarding code definitions, themes or the formulation of CMO configurations were resolved through consensus-based discussions during regular peer-debriefing sessions involving all co-authors. This iterative process allowed for the continuous refinement of the analytical framework and ensured that the findings remained grounded in the empirical data.

#### 3.6.2. Construction of Theme–CMO Configuration Pairs

Themes were systematically linked to CMO configurations in order to articulate how contextual conditions activate mechanisms that generate outcomes. In line with realist-oriented conventions (Pawson & Tilley, 1997), context was operationalised as pre-existing structural, organisational, or personal conditions (e.g., staff continuity, rural geography); mechanism as the response or reasoning elicited by the context in the actor (e.g., reduced anticipatory stress, reliance on procedural memory); and outcome as the reported or observed consequence of that mechanism. In cases where documentary, interview, and observational data diverged within a given configuration, such discrepancies were explicitly discussed by the research team rather than resolved through a simple majority interpretation. In seven instances, this process led to narrowing the scope of the configuration or reformulating it so that it reflected only those conditions under which the mechanism was supported by more than one data source, which constituted a basic requirement for further analytical work with the CMO configuration. This approach integrates thematic analysis with realist-informed analytical logic (Pawson & Tilley, 1997). To enhance the clarity of the interpretation, the mechanisms were also considered from a behavioral perspective, with particular attention focused on how the observed characteristics of the system might influence the decision-making demands on older adults, perceived predictability, and everyday coping processes.

To strengthen the robustness of the audit trail, we actively sought negative cases—instances where proposed mechanisms did not activate or where care arrangements failed to produce expected outcomes. This involved examining projects where integration failed to reduce organizational burden or where environmental cues remained ambiguous for users. These contradictory data points were used to refine the ‘Context’ (C) component of the CMO configurations, identifying the specific systemic prerequisites necessary for successful mechanism activation.

In the final stage of analysis, the authors synthesized the 24 CMO configurations into seven support pillars. This conceptual consolidation was cross-verified by all co-authors to ensure that the higher-level abstraction accurately reflected the functional synergies observed in the field data.

#### 3.6.3. Triangulation

Data triangulation across documents, interviews, and observations was used to enhance the credibility and robustness of findings (Flick, 2023; Denzin, 1978).

Within the data triangulation process, we carefully distinguished between findings derived from contemporary documentary records (from 2000–2024) and retrospective accounts provided by stakeholders in 2025. This approach allowed us to account for potential biases arising from memory decay and to ensure that the long-term institutional trajectory was reconstructed on the basis of evidence rather than present-day idealisations.

#### 3.6.4. Reflexivity

A reflexive approach was maintained throughout the entire research process by all researchers to account for potential researcher bias and positionality (Finlay, 2002). The researchers maintained a reflexive stance, acknowledging the principal author’s ‘insider’ position as a doctoral candidate deeply embedded in the regional context. While this position facilitated access to long-term documentary archives and fostered trust with interviewees, it necessitated a rigorous audit trail. The principal author upheld a strict analytical distance grounded in a realist approach, in order to mitigate the influence of prior, potentially biasing negative or positive experiences and theoretical expectations. To counter potential bias, the analysts systematically compared retrospective parts of interview accounts with documentary evidence and explicitly sought negative cases where proposed mechanisms did not operate. To prevent the findings from merely reproducing the initial theoretical framework, the analyst systematically tested emerging CMOs against contradictory documentary records and stakeholder opinions.

### 3.7. Ethical Considerations

Ethical approval was obtained from the institutional review board (Reference Code: UKFHS/622586/2024; No. 042024/Ren). All participants provided informed consent. Data were anonymised and stored securely in accordance with GDPR. Given the small size of the microregion, particular attention was paid to preventing deductive disclosure (Kaiser, 2009).

## 4. Results

### 4.1. Theoretical Analysis and Domains Linking AiP, HCA, and Integrated Care

The Section 4.1, structured in accordance with the analytical workflow of the study (see Figure 1), corresponds to Step 1 (“Theoretical Framing and Domain Specification”). It reports the results of a theoretical examination of the interrelationships among Aging in Place (AiP), Healthy Cognitive Aging (HCA), and Integrated Care (IC), and identifies the domains linking these concepts. Each domain captures a key area through which integrated care is expected to support ageing in place and cognitive health, drawing on the theoretical foundations outlined in Section 2 (Valentijn et al., 2013; Stern et al., 2019).

#### 4.1.1. Theoretical Interrelations Among AiP, HCA, and IC

The theoretical analysis resulted in the identification of distinct analytical roles of the three concepts. Aging in place was conceptualised as a normative and relational outcome, while healthy cognitive aging was utilized as a theoretical and interpretive lens to examine the supportive capacity of the environment. Integrated care was then explored as a moderating framework that may align these two. The analysis indicates that AiP and HCA are theorised to be mutually reinforcing but not automatically aligned. Cognitive health is theorised to function as a prerequisite for ageing in place, while environmental and service-related conditions characteristic of AiP are theorised to shape cognitive trajectories (Lawton & Nahemow, 1973; Livingston et al., 2020). Integrated care emerges as a moderating concept that may align these processes by enabling mechanisms such as reduced cognitive load, strengthened social embeddedness, enhanced orientation and predictability, and supported autonomy. These mechanisms correspond to those identified in cognitive and environmental gerontology (Stern et al., 2019; Rowles & Bernard, 2013).

Conversely, when contextual conditions are unfavourable, integrated care may fail to activate these mechanisms, limiting its capacity to support either AiP or HCA. This conditionality reflects a realist understanding of causation, where outcomes depend on context–mechanism interactions (Pawson & Tilley, 1997).

#### 4.1.2. Analytical Domains Derived from the Theoretical Examination

Based on this analysis, five analytically defined domains were identified. Definitions and operationalisation of these analytic domains are summarised in Table 2.

These domains reflect key mechanisms identified in the literature on integrated care and cognitive aging (Valentijn et al., 2013; Livingston et al., 2020) and provided a structured framework for subsequent empirical analysis (see Figure 1).

### 4.2. Categorisation of Programme Projects According to Their Relevance to Domains

Project categorisation was guided by the five analytical domains derived from the theoretical examination. These domains operationalise the expected pathways through which integrated care moderates the relationship between AiP and HCA.

To enhance the credibility and minimize subjectivity of the screening process, the classification of the 43 projects into categories A–D was performed independently by two researchers (L.S., A.B.). Any discrepancies in categorization were resolved through peer debriefing all team members until a consensus was achieved.

Projects assigned to Category D demonstrated the highest relevance to these domains. They explicitly addressed continuity of care, multi-level integration, social support, and mechanisms supporting autonomy and shaping conditions relevant to cognitive functioning. This is consistent with theoretical expectations regarding the role of coordinated and person-centered care in reducing system complexity and cognitive burden (Valentijn et al., 2013; Greer et al., 2019).

The screening process identifies Category A and B projects as critical components of the regional care continuum, ensuring macro-systemic stability and physiological stabilization during clinical crises. While these institutional settings are essential for medical safety, they prioritize clinical over ecological continuity, which marks the functional boundary of their role in supporting aging in place (AiP) (Category A, 1 project; Category B, 9 projects). However, by removing older adults from their familiar environments, these interventions may, in certain instances, induce spatial disorientation. Similarly, localized medical outpatient clinics enhance regional healthcare access but do not typically intervene directly in the immediate architecture of the home environment. Consequently, their capacity to modulate the daily behavioral routines that couple AiP and HCA appears relatively limited (Category C, 8 projects).

Projects Category D (25 projects), field-based, domestic, or decentralized community services, are interpreted as modalities that may function as an active environmental support. They demonstrate the potential to reduce executive demands, reinforce environmental predictability, and contribute to the stabilization of behavioral routines within the individual’s micro-environment. This specific subset constitutes the empirical and analytical core of the realist evaluation.

The classification of Programme projects into categories (A–D) is shown in Table 3. This comprehensive catalogue contains all 43 projects implemented within the program framework. Each project is categorized (A–D) based on its alignment with the analytical domains, supplemented by formal academic justifications. The Category D subset selected for deep qualitative analysis. The criteria for classifying projects (A–D) can be found in Table 4.

### 4.3. Thematic Patterns Identified in Project Documentation and Theme–CMO Pairs

Documentary materials from Category D projects were analysed using inductive thematic analysis (Braun & Clarke, 2006). The analysis identified recurring patterns illustrating how integrated care arrangements may support ageing in place and healthy cognitive aging.

Across the analysed projects, themes were examined across five interrelated domains identified in Step 1 of the workflow. These domains correspond to mechanisms identified in prior research on aging environments and cognitive functioning (Livingston et al., 2020; Cacioppo & Hawkley, 2009).

The findings indicate that integrated care contributes not through isolated interventions, but by shaping the broader conditions of everyday life—particularly by stabilising care trajectories, reducing cognitive burden, and supporting relational continuity. Recurring outcomes included sustained ability to remain at home, preserved autonomy, reduced crisis-driven transitions, and improved cognitive and emotional stability. These findings were synthesised into context–mechanism–outcome (CMO) configurations (Pawson & Tilley, 1997), which specify how outcomes emerge under particular contextual conditions. Together with their corresponding themes, they formed theme-CMO pairs. During the iterative refinement and team discussions, five initially identified theme–CMO pairs were excluded. The CMO configurations within these pairs were found to function as operational enablers rather than generative mechanisms directly influencing the HCA and AiP. The final CMO configurations are listed in Table 5.

### 4.4. Refinement of Theme–CMO Pairs via Stakeholder Interviews

To validate, challenge, and refine the preliminary theme–CMO pairs derived from the initial documentary analysis (Step 3), we integrated primary qualitative data from key actors. This step (Step 4) focused on the insights gathered from 14 semi-structured interviews conducted between February and June 2025 (see Table 1). In analyzing these narratives, a clear distinction was maintained between evidence derived from documentary materials of projects (2000–2024) and retrospective stakeholder accounts gathered in 2025. This allows for a nuanced interpretation that accounts for potential recall bias while tracing the program’s long-term evolution.

The stakeholders’ narratives provided necessary empirical depth, transforming abstract document data into lived realities. Specifically, the interviews allowed us to verify whether the hypothesised ‘Mechanisms’ were actively triggered by the ‘Context’ of IC, and whether they yielded the expected ‘Outcomes’ for AiP and HCA. The stakeholder reflections on operational barriers and successes directly shaped the refinement of the initial thematic pairs.

Interview findings confirmed and enriched mechanisms identified in documentary analysis by incorporating actor perspectives and experiential dimensions (Kvale & Brinkmann, 2009). Mechanisms such as continuity, autonomy, and coordination were shown to depend on how actors interpret and engage with care arrangements, highlighting their context-dependent nature.

Participants’ accounts suggest that these arrangements altered how they interacted with the system, particularly by making decisions more straightforward and interactions more predictable. For instance, one participant noted (Informal Caregiver, IC2): “Once all became more coordinated and more connected, I didn’t have to keep checking who to call or what to arrange next. Things became more predictable.”

Participants also described earlier arrangements as involving uncertainty about responsibilities, repeated contacts with multiple providers, and delays in coordinating services.

In behavioral terms, this stabilisation may reduce the need for active system navigation, repeated decision-making, and ongoing adaptation to changing conditions.

This is consistent with participant reflections on earlier arrangements without case manager, where one respondent noted (Municipal Leader, ML1): “Before, each service had to be contacted separately, and it was often unclear who would take responsibility. It required a lot of coordination on our side.”

In contrast, the integrated system with case manager was described as “more straightforward, with fewer steps and clearer responsibilities,” suggesting a reduction in the effort required to manage everyday care arrangements; “…everything was already organised—we knew who would come and when. Before, we had to start calling around and figure it out ourselves”. Participants also recalled situations where fragmented arrangements uncertainty, such as unclear responsibilities or delayed service provision, requiring created additional effort and repeated coordination.

These examples illustrate how coordination at the systemic level is perceived as reducing complexity and increasing predictability at the individual level; how coordinated measures reduce the effort required to navigate the system and support more stable daily routines; and how changes in the organization of the system have made decision-making less demanding.

### 4.5. Empirical Enrichment via Participant Observations

To complement the interview data and mitigate potential self-reporting biases, Step 5 utilised participant observations (N = 11). These observations served to stakeholders’ accounts with real-time operational practices and environmental contexts.

By observing multidisciplinary team meetings, municipal planning sessions, and social activation activities for older adults, we captured subtle, unarticulated dynamics that official documents and formal interviews could not fully register. This observational data enriched our understanding of the contextual preconditions (e.g., rural infrastructure limitations, face-to-face communication habits) and micro-mechanisms (e.g., informal trust-building among staff).

Observational data provided an additional layer of validation by examining how these mechanisms operate in practice (Spradley, 1980). Observations revealed that mechanisms such as continuity and coordination are contingent upon organisational stability, time availability, and professional discretion.

These findings reinforce a realist perspective interpretation: mechanisms are not inherent properties of interventions but are activated only under specific contextual conditions (Pawson & Tilley, 1997).

These patterns are reflected across both documentary materials and interview data, which consistently describe how the stabilisation of care arrangements reduces the effort required to navigate services and maintain everyday routines.

### 4.6. Final Context–Mechanism–Outcome Configurations and Support Pillars

The systematic synthesis of data from the preceding five analytical steps culminated in the formulation of the final Context–Mechanism–Outcome (CMO) configurations. This step represents the ultimate integration of the theoretical frameworks (STAC, cognitive reserve, and brain maintenance) with the empirical realities of the 25-year-old integrated care program.

As shown in Table 5, a total of 24 distinct CMO configurations were finalised. These configurations do not operate in isolation; rather, they are clustered across 7 core support pillars that collectively moderate and facilitate healthy cognitive ageing in place (HCA and AiP) within the microregion as synthesised in Table 6.

The following sections illustrate how specific contextual backdrops trigger mechanisms may support conditions relevant to positive residential and cognitive well-being.

#### 4.6.1. Final CMO Configurations

The qualitative analysis of the 25 Category D projects—drawing upon retrospective documents, 14 stakeholder interviews, and 11 participant observations—indicates 24 specific interaction patterns. These patterns illustrate how distinct environmental contexts may interact with the psychological and cognitive dynamics of older adults.

In the following overview, the final CMO configurations are presented according to the original five domains. It is important to note that at this stage, the domains serve purely as a legacy organizational structure for sorting and referencing the initial framework, as the primary analytical focus has shifted toward the synthesized pillars.

***Domain 1: Environmental stability and continuity of care***
  - CMO 1 (Staff Familiarity): In the context of a rural microregion, long-term personnel stability among formal home care providers appears to offer a stable relational framework. This continuity may support the activation of procedural memory and interpersonal familiarity, which seems to contribute to maintaining daily domestic routines and potentially mitigating behavioral symptoms associated with cognitive decline.
  - CMO 2 (Temporal Predictability): Establishing fixed, relatively predictable schedules for home care visits reinforces external temporal structuring. This environmental predictability appears to compensate for impaired time orientation, potentially enhancing a sense of ontological security and contributing to the stabilization of independent domestic tenure.
  - CMO 3 (Geographic Anchoring): Providing small-scale, decentralized community housing within close proximity to the original place of residence helps preserve local spatial anchoring. This proximity seems to support social orientation, thereby potentially reducing the risk of relocation trauma or confusion associated with moving.
  - CMO 4 (Clinical Reassurance): The continuous presence of professional nursing staff within localized residential social facilities helps create a stable clinical infrastructure. This proximity may foster a sense of reassurance and relational trust, which is often linked to proactive health stabilization and a potential reduction in disruptive emergency hospitalizations.
  - CMO 5 (Institutional Permanence): The 25-year history of the voluntary association of municipalities offers a long-term framework of structural support. This continuity appears to cultivate systemic trust and existential security among families, which may facilitate proactive decision-making regarding long-term aging-in-place strategies.
  - CMO 6 (Care Convergence): The concurrent delivery of standard home care and specialized domestic hospice services creates an integrated support environment. This arrangement seems to alleviate caregiver burden and emotional distress, potentially facilitating a more seamless and dignified end-of-life transition within the home.
***Domain 2: Coordination and integration across health and social care***
  - CMO 7 (Transition Management): Formalized multidisciplinary case conferences and cross-sectoral transition protocols serve as an administrative safety net. This coordination attempts to shift a substantial portion of the informational burden away from the family, which may help maintain continuity of care during potentially high-risk hospital discharges.
  - CMO 8 (Inpatient Integration): Embedding specialized social work directly within acute hospital wards facilitates early planning for home environmental modifications. This proactive approach appears to encourage predictable discharge pathways, which may contribute to lowering the risk of readmission.
  - CMO 9 (Information Sharing): The structural linkage between specialist outpatient clinics and home healthcare services, supported by shared documentation, aligns clinical insights. This transparency may limit diagnostic latency and redundant examinations, which appears beneficial for clinical safety and the potential reduction in executive stress.
  - CMO 10 (Unified Navigation): A centralized referral system and shared intake pathways managed by the municipal association facilitate access to services. This alignment aims to mitigate system fragmentation, attempting to create a clearer entry point into the local care ecosystem.
***Domain 3: Social embeddedness and relational support***
  - CMO 11 (Field Mobilization): Regular home visits combined with the mobilization of local volunteers help establish proactive social contact. This presence may assist in activating latent community solidarity and affective ties, which is often observed to alleviate feelings of loneliness and isolation.
  - CMO 12 (Civic Activation): Accessible community gardens and social activation centers within the microregion provide opportunities for engagement. These spaces may stimulate valued social roles and mutual support, which appears to be a factor in decelerating cognitive withdrawal and potentially enhancing subjective well-being.
  - CMO 13 (Natural Surveillance): The context of small, cohesive village communities allows for the utilization of informal neighborhood networks. This proximity facilitates discrete situational monitoring and may function as an informal early warning network for subtle cognitive or physical changes.
***Domain 4: User autonomy and participation***
  - CMO 14 (Resource-Oriented Planning): Co-creating individualized care plans focused on preserved functional capabilities supports user competence. This approach accounts for the subjective autonomy of the older adult, which seems to help prevent the development of learned helplessness and passivity.
  - CMO 15 (Validating Communication): Training staff in flexible techniques of validating communication supports respect for individual daily routines. This approach appears to reinforce relational autonomy and dignity, which typically correlates with a greater willingness to accept services.
  - CMO 16 (Environmental Mastery): A regional assistive technology rental service paired with in-home training adapts the immediate physical surroundings. This supportive framework appears to enhance functional self-efficacy, potentially supporting prolonged independent living.
  - CMO 17 (Participatory Inclusion): Structurally involving older adults and their representatives in program feedback mechanisms respects the user’s voice. This participation seems to promote a sense of belonging and empowerment, which may enhance the long-term legitimacy of the program.
***Domain 5: Cognitive load, stress, and orientation***
  - CMO 18 (Single Point of Entry): A single, clearly identifiable coordination center simplifies system navigation. This structural anchor attempts to absorb a significant portion of the complex logistical burden, which may mitigate the risk of executive overload or administrative distress.
  - CMO 19 (Practical Proxy Support): Direct, practical assistance with household management and administrative tasks helps compensate for declining cognitive capacities. This intervention aims to reduce demands on working memory, serving as a factor that may prevent financial vulnerability and support independent tenure.
  - CMO 20 (Spatial Simplification): Implementing intuitive visual cues and architectural simplification in local facilities helps reduce environmental stress. These modifications focus on minimizing spatial ambiguity, which seems to favorably influence independent mobility and may assist in reducing fall risks.
  - CMO 21 (Cognitive Exercises): Structured memory training integrated into daily life via familiar micro-tasks embeds cognitive stimulation within standard routines. This practice is oriented toward stimulating cognitive reserve, which, in certain cases, may help slow the rate of functional decline in the early stages of cognitive impairment.
  - CMO 22 (Crisis De-escalation): The availability of a 24/7 crisis hotline supported by rapid mobile teams offers an immediate emotional safety net. This accessibility may significantly contribute to mitigating acute panic, appearing as an important element in preventing severe behavioral decompensation.
  - CMO 23 (Caregiver Protection): Accessible in-home respite services and targeted counseling for informal caregivers help protect the family environment. This support aims to reduce caregiver burnout, which appears to stabilize the informal caregiving structure and may help delay permanent institutionalization.
  - CMO 24 (Tactile Activation): Targeted volunteer initiatives, including canine-assisted interventions, offer non-pharmacological support. These sessions activate non-verbal communication channels and tactile emotional responses, which are frequently linked in the data to a temporary reduction in manifestations of agitation or apathy.

All CMO final configurations can be found in Table 5.

#### 4.6.2. From CMO Final Configurations to Core Support Pillars

The final phase of the synthesis involves a systematic transition from localized field data to higher-level theoretical abstraction. Instead of presenting the 24 context-mechanism-outcome (CMO) configurations as isolated operational patterns, they were thematically grouped and synthesized into seven fundamental pillars based on the functional and generative characteristics of their underlying mechanisms. This conceptual consolidation captures how micro-level operational routines are absorbed into macro-level systemic and organizational structures. These seven pillars provide an integrated, behaviorally grounded explanatory framework that illustrates how the long-established community care ecosystem functions as a conditional moderator—by structuring external contexts to activate latent capacities relevant both to aging in place (AiP) as well as for healthy cognitive aging (HCA), which corresponds to the explicit formulations expressed in the study’s conclusion.

The research process itself prompted a shift in how the final findings are presented. Based on the insights gained during data triangulation and the collective experience of the research team, it was deemed more appropriate to move beyond the initial organizational grid of domains. To present the conclusions in a more comprehensible and communicable form, the 24 CMO configurations were sorted into seven core support pillars. This iterative grouping process allowed us to move from a fragmented thematic view to a holistic explanatory framework that better represents the practical reality of the integrated care ecosystem. This conceptual consolidation was carried out in such a way that individual CMO configurations were either chosen as representative for a given core pillar (based on their logical primacy, systemic scale, or thematic breadth within the data) or were absorbed into a given pillar. In this final stage, the research team transitioned from the initial organizational grid (domains) to a more integrated and communicable explanatory structure. This consolidation was an iterative process conducted with the participation of all team members, focusing on identifying the Core Representative CMOs that define the essence of each pillar. Through an iterative process the 24 final CMO configurations were grouped into seven support pillars. This process involved identifying ‘Core Representative CMOs’ and clustering ‘Supporting/Absorbed CMOs.’ The process originally began with five pillars; however, through repeated team discussions and empirical refinement, it became evident that regrouping into seven pillars was necessary to accurately represent the data. This shift allowed for a clearer distinction of ‘Pillar 1: Institutional and Governance Bedrock’ as the absolute prerequisite upon which the operational dynamics of the remaining pillars depend. This transition from field data to theoretical abstraction reflects the cumulative epistemological trajectory of the study. While the initial five analytical domains were derived strictly from a theoretical examination of the interrelationships between AiP, HCA, and IC, they were subsequently operationalized into preliminary theme–CMO pairs. Through empirical confrontation with retrospective documents, stakeholder interviews, and participant observations, these pairs were refined, and the contextual boundaries were deepened. By systematically discarding the operational narrative themes to extract the underlying causal mechanisms, the finalized 24 CMO configurations were established. Consequently, the resulting seven pillars do not merely replicate the baseline theory; rather, they represent a richer, empirically validated framework where abstract concepts have been refined through real-world practice, rendering the final synthesis potentially transferable to analogous regional settings. To demonstrate the empirical grounding of the synthesized pillars, we provide representative illustrations for each core CMO configuration. These examples have been purposively selected from the extensive 25-year longitudinal corpus and qualitative data set to serve as prototypical instances of patterns observed repeatedly across multiple projects and stakeholder accounts. For reasons of conciseness, only one illustrative case per pillar is presented here, though each represents a recurring thematic configuration identified during the triangulation process.

A structural symmetry exists between the initial theoretical domains and this finalized synthesis of pillars. The operational dynamics of Domain 1 (Environmental stability and continuity of care) naturally branched and matured into Pillars 1, 2, and 3, whereas Domains 2 through 5 correspond directly to Pillars 4 through 7, respectively.

Within this consolidated architecture, Pillar 1 occupies a unique epistemological position: unlike the subsequent pillars, Pillar 1 represents the overarching institutional meta-condition of the entire ecosystem. The 25-year history of the voluntary association of municipalities provides the structural permanence, legal stability, and cross-municipal trust that constitute a prerequisite; without this foundational bedrock, the specific context-mechanism dynamics within the remaining six pillars could not be triggered or sustained.

The pillars that emerged from this conceptual consolidation are as follows:

***Pillar 1: Institutional and Governance Bedrock***
  - Core Representative CMO: CMO 5 (Institutional Permanence/25-year history of the voluntary association of municipalities).
  - Supporting/Absorbed CMO: None (stands as the baseline structural context).

The following case serves as a representative illustration of how institutional permanence (CMO 5) functions as a systemic anchor:

- **Documentary Excerpt:** ‘Strategic Development Plan: The voluntary association of municipalities formally declares a long-term commitment to the solidarity-based funding of integrated services… ensuring structural permanence regardless of external grant cycles.’
- **Interview Quotation (ML1, Municipal Leader):** ‘In our region, integrated care is not a matter of debate every election. The structure has existed for over twenty years… This systemic stability fosters deep community trust.’
- **Observation Note:** During a board meeting, mayors reach a consensus on increasing the operational budget, demonstrating political alignment acting as an absolute prerequisite for the ecosystem.

***Pillar 2: Relational Continuity and Memory Scaffolding***
  - Core Representative CMO: CMO 1 (Staff Familiarity/long-term personnel stability reducing anxiety).
  - Supporting/Absorbed CMOs: CMO 4 (Clinical Reassurance/embedded nursing staff), CMO 6 (Care Convergence/integrated standard and domestic hospice care).

The activation of procedural memory through relational continuity (CMO 1) is evidenced by the following typical interaction:

- **Documentary Excerpt:** ‘Care Provider Manual: To minimize cognitive anxiety… coordinators must prioritize assigning a consistent team of a maximum of two regular caregivers to each household.’
- **Interview Quotation (OA1, Older Adult):** ‘She has been coming to see me for years. She knows exactly how I like my tea… I don’t have to explain anything, which makes me feel much more at ease.’
- **Observation Note:** A caregiver enters a client’s home using a familiar greeting; the client responds with immediate recognition and calmness, indicating that the stable presence activates interpersonal familiarity.

***Pillar 3: Environmental and Temporal Predictability***
  - Core Representative CMO: CMO 2 (Temporal Predictability/fixed visit schedules compensating for time disorientation).
  - Supporting/Absorbed CMOs: CMO 3 (Geographic Anchoring/small-scale decentralized housing safeguarding local spatial ties).

The compensatory role of temporal predictability (CMO 2) in managing cognitive disorientation is demonstrated by this representative instance:

- **Documentary Excerpt:** ‘Individualized Care Plan: Home care visits are strictly scheduled for 08:30 (morning hygiene) and 12:00 (meal delivery) daily to provide a stable temporal structure.’
- **Interview Quotation (PC2, Professional Caregiver):** ‘He is always waiting by the window ten minutes before I arrive. If I were late, it would cause him unnecessary stress.’
- **Observation Note:** A senior has his medication ready on the table exactly five minutes before the scheduled visit, demonstrating how fixed schedules help compensate for impaired time orientation.

***Pillar 4: Cross-Sectoral Coordination***
  - Core Representative CMO: CMO 7 (Transition Management/multidisciplinary case conferences and transition protocols).
  - Supporting/Absorbed CMOs: CMO 8 (Inpatient Integration/specialized hospital social work), CMO 9 (Information Sharing/shared clinical and social documentation), CMO 10 (Unified Navigation/centralized intake pathways).

The elimination of administrative silos during high-risk transitions (CMO 7) is reflected in this prototypical account:

- **Documentary Excerpt:** ‘Inter-sectoral Cooperation Agreement: Health and social care departments utilize a unified system… automatically triggering a multidisciplinary assessment of needs.’
- **Interview Quotation (CM1, Case Manager):** ‘Families call our center, and we coordinate whether a nurse or a social worker needs to visit first. The client is shielded from the burden of navigating agencies.’
- **Observation Note:** During a multidisciplinary case conference, a GP, a home care supervisor, and a social worker jointly plan a transition, ensuring a seamless web of care that prevents functional fragmentation.

***Pillar 5: Socio-Relational Embeddedness***
  - Core Representative CMO: CMO 11 (Field Mobilization/regular home visits and volunteer activation).
  - Supporting/Absorbed CMOs: CMO 12 (Civic Activation/community gardens and social centers), CMO 13 (Natural Surveillance/informal neighborhood monitoring networks).

The function of the community as a relational buffer (CMO 11) is illustrated by this characteristic field example:

- **Documentary Excerpt:** ‘Volunteer Management Methodology: Volunteers are trained as social partners focusing on preventing isolation among home-bound seniors through regular social contact.’
- **Interview Quotation (SW1, Social Worker):** ‘I visited him with the volunteer to chat about village news. It helps him feel like he is still a part of the community, even though he can no longer leave his house easily.’
- **Observation Note:** A volunteer and a senior sit together on a porch observing the village activity; this informal interaction serves as a social anchor that mitigates chronic loneliness.

***Pillar 6: Supported Autonomy and Agency***
  - Core Representative CMO: CMO 14 (Resource-Oriented Planning/individual plans centered on preserved capabilities).
  - Supporting/Absorbed CMOs: CMO 15 (Validating Communication/flexible validating techniques), CMO 16 (Environmental Mastery/assistive technology rentals and in-home training), CMO 17 (Participatory Inclusion/structural user feedback mechanisms).

The shift from paternalistic care to agency protection through resource-oriented planning (CMO 14) is captured here:

- **Documentary Excerpt:** ‘Individualized Assessment Form: Priority is given to mapping the client’s preserved capabilities… which the service then scaffolds to support maximum autonomy.’
- **Interview Quotation (SW1, Social Worker):** ‘If a client insists on going for the mail, we don’t do it for them. We provide the support to make that walk safe. We don’t take over their life; we scaffold it.’
- **Observation Note:** A caregiver waits patiently for the senior to fasten their own buttons, only handing them pieces of clothing; this reinforces the client’s internal locus of control and residual capacities.

***Pillar 7: Reduction In Cognitive and Navigational Burden***
  - Core Representative CMO: CMO 18 (Single Point of Entry/centralized coordination center absorbing system complexity).
  - Supporting/Absorbed CMOs: CMO 19 (Practical Proxy Support/household and administrative assistance), CMO 20 (Spatial Simplification/intuitive architectural and visual cues), CMO 21 (Cognitive Exercises/memory training embedded in familiar micro-tasks), CMO 22 (Crisis De-escalation/24/7 hotline and rapid mobile teams), CMO 23 (Caregiver Protection/in-home respite care and counseling), CMO 24 (Tactile Activation/volunteer-led canine-assisted activities).

The absorption of systemic complexity through a structural anchor (CMO 18) is evidenced by this representative evidence bundle:

- **Documentary Excerpt:** ‘Information Leaflet: One Door for All Needs. The center handles all benefits applications, medical coordination, and service scheduling on behalf of the client.’
- **Interview Quotation (IC2, Informal Caregiver):** ‘Instead of filling out ten forms, I came here, signed one power of attorney, and they handled all the bureaucracy. It saved me from complete burnout.’
- **Observation Note:** A social worker sits with a senior, helping them organize official mail into clear, color-coded folders; this intervention absorbs the navigational burden and prevents care environment collapse.

A synthesis of the finalized CMO configurations into key supporting pillars is contained in Table 6.

### 4.7. Conceptual Synthesis: Integrated Care as a Conditional Moderator

**Figure 2:**
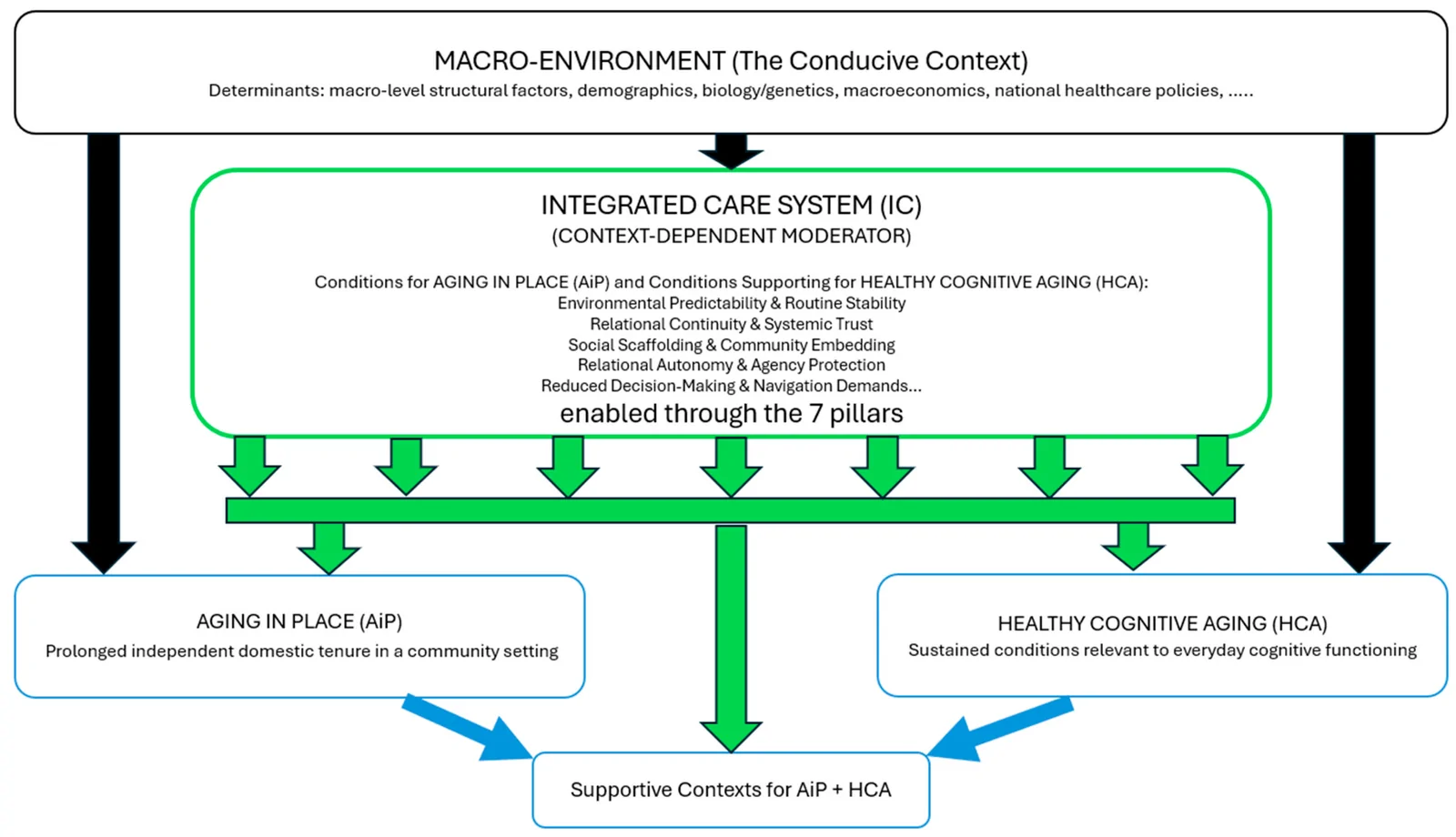
Conceptual and Ecological Framework of the Study. The diagram illustrates Aging in Place (AiP) and Healthy Cognitive Aging (HCA) as ontologically independent, theoretically mutually reinforcing dynamics operating within a shared macro-environment. Integrated care (IC) is conceptualized as an adaptive, conditional context-dependent moderator that can mitigate external environmental complexities and systemic demands, support environments conducive to Healthy Cognitive Ageing (HCA) and Aging in Place (AiP), enabling conditions and governance structures that are theorised to jointly support Aging in Place (AiP) and conditions relevant to Healthy Cognitive Ageing (HCA).

The empirical findings of this study are synthesized into an overarching conceptual model. This framework positions the municipal integrated care program as a context-dependent, conditional moderator that reconfigures the relationship between a vulnerable individual and their domestic environment. This system configuration is visualized in Figure 2.

As conceptualized in Figure 2, the empirical reality of the study may be effectively captured through an ecological framework of shared environments rather than a linear causal chain. Aging in Place (AiP) and Healthy Cognitive Aging (HCA) possess their own ontologically independent dynamics and can inherently exist outside formal support systems. Both these concepts, alongside the integrated care (IC) program (here operationalized as the municipal program Programme), operate within the same overarching macro-environment characterized by regional socioeconomic conditions, biological health trajectories, and municipal policies.

Within this shared space, the integrated care program functions uniquely as a conditional moderator. As a theoretical lens, it does not directly produce AiP or HCA; instead, it acts as an adaptive filter or buffer shaping the organizational and social conditions relevant to them intercepts the raw complexities of the external environment. When properly activated under conducive contextual conditions—such as the region’s historical institutional trust—the IC system translates macro-resources into a stabilized micro-environment for the individual. By generating specific, shared mechanisms (environmental stability, relational continuity, and reduced cognitive load), the program shields the older adult from systemic complexity. Crucially, this moderation is theorised not merely to support AiP and conditions relevant to HCA as isolated parallel targets, but to safeguard and amplify a reciprocal, synergistic feedback loop between them; this remains a conceptual proposition rather than an empirically demonstrated effect, since cognitive functioning was not directly measured.

Figure 2 conceptualises integrated care as a context-dependent moderator: a municipal program can contribute to ageing in place and to conditions theorised to be relevant to healthy cognitive ageing by activating enabling conditions in conducive contexts, together with determinants that lie beyond integrated care.

The conceptual architecture illustrated in Figure 2 operates across three interrelated dimensions:

- The Conducive Context: The long-term sustainability of the system is conditioned by a specific macro-context characterized by more than two decades of stable municipal leadership, the voluntary pooling of local resources, and an established institutional trust. This historical and political background forms the necessary baseline that allows the micro-level care arrangements to function continuously.
- The Activation of Enabling Conditions: Rather than acting as a direct intervention, the integrated care program functions as a filtering layer that is theorised to help buffer external complexities and contributes to translating into specific, tangible enabling conditions within the older adult’s immediate surroundings. By stabilizing care environments, securing relational continuity, and reducing everyday decision-making demands, the program contributes to decompressing the micro-environment It creates an external compensatory framework that may mitigate the risk of functional failure; however, this remains a conceptual proposition rather than an empirically demonstrated outcome, since cognitive functioning was not directly measured.
- The Conceptual Interdependence of Home and Cognition: The model proposes that Aging in Place (AiP) and conditions relevant to Healthy Cognitive Aging (HCA) are not isolated parallel outcomes, but are theorised to be mutually reinforcing. This is conceptualised as a safe, predictable relational space in which everyday behavioural routines can unfold with reduced anxiety and, potentially, reduced pressure toward premature institutionalisation; this remains a theoretical proposition rather than an empirically demonstrated outcome, since cognitive functioning was not directly measured.

The framework acknowledges the open nature of community care by explicitly accounting for external determinants. Factors such as primary biological disease progression, family dynamics, or macroeconomic policy shifts continue to interact with the system from the outside, highlighting that integrated care acts as a conditional moderator rather than a closed, absolute determinant. In this framework, IC provides the necessary scaffolding that enables the synergy between home and cognition.

The conceptual model must also account for internal contextual prerequisites. The observed outcomes were facilitated not only by the IC framework itself but also by a bedrock of effective governance and regional solidarity. Without the exceptional commitment of the pioneering professionals and the manageable scale of the microregion, which allowed for rapid and informal coordination, the activation of certain mechanisms—such as unified navigation or crisis de-escalation—might have been significantly more constrained.

## 5. Discussion

### 5.1. Epistemological Reflection and Theoretical Integration of Neurocognitive Frameworks

Before deconstructing the systemic implications of our findings, a crucial epistemological and methodological clarification must be articulated regarding the operationalization of the cognitive aging frameworks introduced in the literature review. Throughout the multi-method data collection phase, all members of the research team possessed deep, granular knowledge of Nyberg’s Brain Maintenance theory (Nyberg et al., 2012) and the Scaffolding Theory of Aging and Cognition (STAC) (Park & Reuter-Lorenz, 2009). Given that this study is a qualitative, realist-informed case study grounded in document analysis, stakeholder interviews, and participant observations, direct empirical verification or refutation of neurobiological parameters (such as neural integrity or compensatory neural recruitment) lay strictly beyond our empirical scope. Consequently, explicit mentions of these neurobiological terms were naturally absent from the raw documentary records and field notes.

These frameworks nonetheless functioned as sensitizing concepts during fieldwork, shaping the research team’s theoretical attention to phenomena such as routine stability, predictability, and perceived cognitive ease. It is important to be explicit about the epistemic status of this connection: because no direct neurocognitive or psychometric measures were collected, the qualitative material cannot verify, validate, or serve as a proxy for Brain Maintenance- or STAC-related neural processes. What the data can support is a more modest, behaviourally grounded observation: when contextual stability and environmental predictability were associated, in participants’ accounts and in observed practice, with reduced reported cognitive load and preserved everyday functioning, these patterns are consistent with, and may be usefully interpreted through, Brain Maintenance and STAC as theoretical lenses. This should be read as a theoretically informed interpretation offered by the research team, not as an empirical test or validation of these neurocognitive theories, nor as evidence of any specific underlying neural mechanism.

### 5.2. Integrated Care as an Externalized Compensatory Framework

The main theoretical contribution of this study is that we conceptualize integrated care as an active, external compensatory framework that conditions both aging in place (AiP) and healthy cognitive aging (HCA). Traditional frameworks often view independent home-keeping and cognitive preservation as distinct biobehavioral or clinical outcomes that need to be addressed through individualized pharmacological or lifestyle interventions. Our findings are consistent with, and suggest a qualitative extension of, Lawton and Nahemow’s (1973) classic environmental pressure theory: the empirical material indicates that a 25-year localized ecosystem of integrated care may structurally recalibrate the microenvironment and absorb some of the executive and navigational burdens that, according to this theory, typically trigger home-keeping failure in cognitively vulnerable older adults. Lawton’s model posits that an individual’s behavioral and psychological adaptation is a function of the balance between their intrinsic competence and the demands (press) of their environment. As cognitive competence progressively declines due to neurodegenerative processes, an unmitigated environmental press inevitably triggers behavioral decompensation, functional failure, and premature institutionalization. Our empirical analysis indicates that a long-term, localized community care ecosystem can structurally reconfigure and decompress this micro-environment.

Specific environmental arrangements, such as fixed, predictable visitation trajectories or spatial home simplifications, appear, based on stakeholder accounts, to function as external nodes that may compensate for residents’ internal executive function deficits, although this compensatory effect was not directly assessed. These findings extend purely biomedical narratives, such as those presented by Prince et al. (2015), that link autonomous living almost exclusively to an internal neurological state. In alignment with critical gerontological discourses on the fluid and relational meaning of home (Sixsmith & Sixsmith, 2008; Wiles et al., 2012), our findings suggest that the domestic sphere may be reconfigured into a supportive environment that, from a theoretical standpoint, could plausibly help offset the practical consequences of working memory deficits and executive dysfunction through structured external scaffolding. As cognitive functioning was not directly measured, this compensatory mechanism should be understood as an interpretation grounded in participants’ accounts rather than an empirically demonstrated effect.

However, it is critical to recognize that the effectiveness of compensatory scaffolding is not uniform across the aging population. As Cocchieri et al. (2025) note, individuals with low health literacy or high nursing complexity are particularly vulnerable to fragmentation of care systems. In our case study, although the integrated system seeks to reduce navigational complexity, the extent to which older adults can make use of these enabling conditions may still be moderated by their baseline ability to understand and engage with care structures. Our findings contribute to bridging the gap between abstract coping processes and empirical reality: in a fragmented system, coping requires high executive function, while the IC system can externalize this burden and translate complex psychological coping into stable daily routines.

### 5.3. Relational Continuity and Psychosocial Dynamics

The identified Pillar 1 (Relational continuity and stabilization of everyday routines) and Pillar 4 (Social embeddedness) resonate strongly with Tom Kitwood’s Person-Centered Approach to Dementia (Kitwood, 1997). Kitwood argued that a substantial proportion of functional and cognitive deterioration in individuals with cognitive impairment is accelerated not by neuropathology alone, but by a malignant social psychology embedded within the care environment (e.g., social isolation, depersonalization, disruptions of familiarity).

Our findings—such as the emphasis on minimizing field staff turnover and the linking of formal care with natural, informal neighborhood supervision— are consistent with, and lend qualitative support to, this framework, although cognitive functioning and the proposed neurocognitive mechanisms were not directly measured. By ensuring interpersonal predictability, the program appears, based on stakeholder accounts, to reduce the activation of anxiety and neophobia, which are understood to exacerbate behavioral and psychological symptoms of dementia (BPSD) (Murman et al., 2002). In contrast to fragmented care environments where older adults face a constantly changing mix of unfamiliar care professionals, relational stability may function as a protective moderator that, according to participants’ accounts, could help sustain procedural memory traces and support a sense of individual personhood.

### 5.4. Cross-Sectoral Synergies and Mitigating Transitional Trauma

The cumulative and synergistic nature of the identified pillars corroborates Leutz’s Laws of Care Integration (Leutz, 1999), specifically the axiom that integration must address the entire continuum of human vulnerability rather than isolated medical crises. Current transitional care literature (Naylor et al., 2011; Coleman, 2003) extensively documents the phenomenon of transitional trauma—the profound cognitive shock, disorientation, and subsequent functional decline experienced by vulnerable older adults during uncoordinated handoffs between acute hospital wards and their homes.

As summarized in Table 5, embedding a dedicated program social worker directly into inpatient units alongside formal cross-sectoral transit protocols appears, based on stakeholder accounts, to function as an effective bridge between sectors. This interface may help mitigate institutional bifurcation (the systemic split between health and social care sectors), which remains a paramount barrier to sustainable community-based care globally (Goodwin, 2013; World Health Organization, 2021). By absorbing the informational and logistical friction of transitions, the program is perceived by participants to shield both the senior and informal caregivers from acute stress—a possibility that aligns with systemic goals highlighted by the Lancet Commission on dementia prevention and care (Livingston et al., 2020).

### 5.5. Policy and Practical Implications for Community Care

For policymakers, health system architects, and community gerontologists, this study provides a transferable basis for discussing how to approach demographic aging and dementia prevalence within rural environments.

First, the data suggest that a voluntary association of municipalities can provide a functional institutional foundation. Centralizing administration and funding at the microregional level appears to be one option for bridging the traditional sectoral divide between municipal social services and regional (or state) healthcare systems. This structural alignment facilitates a more cohesive distribution of resources based on local needs.

Second, because only Category D projects were examined in depth in this study, our findings speak directly to the potential value of decentralized, field-based community solutions rather than to a direct empirical comparison with projects Category B (Institutional Healthcare Infrastructure), which was not analysed qualitatively here. Strengthening personnel stability, introducing predictable schedules, and building environmental compensatory frameworks appear, based on our findings on Category D projects, to be potentially effective pathways toward sustaining long-term independent living within the community. Whether this implies that public investment should be reallocated away from inpatient infrastructure is a policy question that this single-case study projects Category D focused design cannot itself answer, and would require a dedicated comparative analysis of Category B projects that falls outside the scope of the present study. Therefore, our findings regarding the benefits of community-based solutions (Category D) should not be interpreted as a direct empirical rejection of institutional clinical infrastructure. The study tentatively indicates that public investments might benefit from achieving a calibrated balance between robust clinical infrastructure (Category B) and the expansion of decentralized behavioral scaffolding (Category D). Our findings suggest that while Category B remains indispensable for acute crises, Category D solutions offer a specialized pathway to address the psychosocial and environmental needs of healthy cognitive aging.

Third, while this case originates in a specific Czech microregional context, several of the underlying mechanisms appear to reflect general organisational-design principles rather than features unique to this Programme, and may therefore be more broadly applicable to other health and social care systems, whereas others are more closely bound to the particular institutional, political, and cultural conditions of this setting.

### 5.6. Study Limitations

Interpreting the findings of this retrospective qualitative realist case study, with a longitudinal documentary component, requires a rigorous acknowledgement of several methodological and contextual limitations. In particular, and consistent with Section 5.1, findings relating to healthy cognitive aging should be read throughout this article as theoretically informed interpretations rather than as directly measured outcomes:

- Single-Case and Microregional Specificity: The empirical data originate exclusively from a single rural microregion in the Czech Republic governed by a unique voluntary association of municipalities. This specific institutional architecture features an exceptionally high degree of long-term political alignment and financial pooling (resource pooling) over more than two decades. Consequently, the transferability of these findings to highly urbanised settings, metropolitan areas, or regions characterised by extreme political fragmentation or heavily privatised, market-driven social care sectors may be structurally constrained. While our analysis attributes these outcomes to the integrated care model, we must acknowledge that the program’s success is inextricably linked to several specific contextual advantages. These include an unusually stable political leadership that remained consistent for over 25 years, shielding the program from electoral disruptions, and a highly favorable funding environment enabled by the voluntary pooling of municipal resources. In a less stable or more fragmented political context, the same integrated care mechanisms might not have achieved comparable longevity or effectiveness.
- Retrospectivity and Data Survival Bias: Documentary analysis spanning a 25-year period (2000–2024/2025) is inherently susceptible to variations in data quality and completeness. Records from the program’s early phases (the early 2000s) were not originally compiled for scientific evaluation. This introduces a risk of data survival bias, where successful initiatives or well-documented outcomes are more likely to have been preserved, while records detailing operational dead-ends, structural failures, or discontinued projects may have been lost or discarded over time.
- Stakeholder Recall Bias: Qualitative insights derived from semi-structured interviews with long-serving key stakeholders and pioneers are bound by the limitations of human memory and retrospective rationalization. Respondents may inadvertently idealize early program milestones, smooth over historical conflicts, or retroactively project contemporary professional standards, guidelines, and expert knowledge onto strategic decisions made two decades prior.
- Absence of Direct Psychometric and Quantitative Proxies: While rich in qualitative and behavioral depth, this evaluation does not correlate the identified Context-Mechanism-Outcome (CMO) configurations with direct, individual clinical data, such as longitudinal Mini-Mental State Examination (MMSE) or Montreal Cognitive Assessment (MoCA) trends. Furthermore, research lacks a quantitative control group of seniors living in identical socio-demographic conditions without program support. It is therefore impossible to exactly isolate the net effect of the program, calculate precise hazards, or quantify the exact number of months institutionalization was delayed.
- Confounding Macro-Systemic and Policy Shifts: Over the 25-year observation window, the broader Czech health and social care systems underwent substantial structural transformations (e.g., the introduction of the landmark Act on Social Services in 2006, shifting regional subsidy frameworks, and evolving healthcare reimbursement models). These macro-contextual variables could not be fully isolated or controlled within a realist micro-level case design, meaning that the activation of certain local mechanisms may have been co-triggered or moderated by external macro-policy shifts.

### 5.7. Transferability of Findings to Other Contexts

Although this study examines a single rural Czech microregion, several of the identified mechanisms appear to reflect general organisational-design principles rather than features specific to this Programme, and may therefore be broadly transferable. These include the reduction in decision-making complexity through a single point of entry and case management (CMO 10, CMO 18), the use of fixed and predictable scheduling to reduce anticipatory stress (CMO 2), the minimisation of staff turnover to preserve relational continuity (CMO 1), and the mobilisation of informal community and volunteer networks alongside formal services (CMO 11, CMO 24). These principles are consistent with Leutz’s Laws of Care Integration (Leutz, 1999) and with broader taxonomies of integrated care (Valentijn et al., 2013), and do not, in principle, presuppose the specific institutional form found in this Programme.

Other elements of the Programme’s success appear more closely tied to the specific Czech and regional context and may be harder to transfer directly. These include the voluntary association of 27 municipalities under a single administrative and budgetary structure, more than two decades of stable political leadership and inter-municipal trust, and a low-turnover workforce embedded in a socially cohesive rural community with dense pre-existing informal networks. In more fragmented, urbanised, or market-driven health and social care systems—where providers compete rather than pool resources, political leadership turns over more frequently, and informal neighbourhood networks are typically weaker—replicating the same institutional pathway may be considerably more difficult, even if the underlying behavioural mechanisms (e.g., reduced navigational burden, relational continuity) remain conceptually relevant.

Furthermore, the relatively small scale of the microregion likely functioned as an alternative explanation for the high degree of inter-professional trust. In a community where providers and municipal leaders share lifelong social ties, the exceptional motivation and commitment of the professionals involved may stem as much from personal relational accountability as from formal organizational protocols. This ‘social capital’—often characteristic of cohesive rural communities—represented a significant non-monetary resource that may be difficult to replicate in larger, more anonymous urban healthcare systems where staff turnover is typically higher and professional relationships are more transactional.

On this basis, we tentatively suggest that successful transfer of this model to other settings would likely require, at minimum: (1) a single accountable coordinating body with a stable, multi-year funding commitment, capable of acting as a functional substitute for the voluntary municipal association described here; (2) organisational policies that actively protect front-line staff continuity; (3) a deliberate strategy for engaging existing community and volunteer networks, particularly in settings where such networks are not already dense; and (4) sustained leadership commitment extending well beyond typical political or budgetary cycles. Where these conditions cannot be met—for example, in large metropolitan systems with high provider fragmentation, high staff turnover, or short-term project-based funding, the mechanisms identified in this study may still offer a useful conceptual template, but the magnitude and consistency of their effects should not be assumed to replicate those observed in this particular 25-year, small-scale case.

## 6. Conclusions

This study examined how ageing in place can be supported through integrated health and social care, with particular attention to healthy cognitive aging. By integrating documentary analysis, interviews, and observations, it developed a set of empirically grounded CMO configurations explaining how integrated care operates in practice.

The findings suggest that the conditions facilitating AiP and HCA may be mutually supported through shared mechanisms, including environmental stability, continuity, social embeddedness, supported autonomy, and reduced cognitive load. Integrated care functions as a conditional moderator: it supports these outcomes only when it activates relevant mechanisms under conducive contextual conditions.

Overall, the study shows that implementing ageing in place through integrated care is neither automatic nor purely technical. It depends on sustaining stable, predictable, and relationally supportive environments that reduce cognitive burden and support agency by shaping stable and predictable conditions relevant to everyday cognitive functioning. These findings provide a theoretically grounded basis for the design, evaluation, and governance of integrated care systems in ageing societies.

These findings can also be interpreted as indicating changes in behavioural conditions, where general coping processes appear to be reflected in reduced decision-making demands, enhanced predictability, and more stable everyday routines. This study contributes by (1) conceptually linking AiP and conditions relevant to HCA through shared, theoretically grounded mechanisms identified in the qualitative data, (2) conceptualising integrated care as a context-dependent moderator, and (3) providing a rare 25-year retrospective qualitative perspective with a longitudinal documentary component.

Integrated care does not directly influence cognitive outcomes; rather, it may shape the conditions under which cognitive functioning could be maintained, and may contribute to reconfiguring the micro-environment, plausibly buffering environmental press and reducing perceived executive load. However, the limits of such implementation are heavily conditional upon systemic prerequisites.

## Figures and Tables

**Table 1:** Overview and Structural Characterisation of Empirical Data Sources. Summary of qualitative and documentary data streams utilized within the retrospective longitudinal case study, including sample volume, key characteristics of stakeholders, specific retrospective documentary collection timeframes (2000–2024), and their respective analytic roles within the Context–Mechanism–Outcome (CMO) heuristic.

Data Source Type	Sample Characteristics & Volume	Data Collection Timeframe	Specific Role Within the CMO Heuristic
Documentary Materials	Strategic plans, project proposals, operational guidelines, minutes from multidisciplinary boards, annual reports, external evaluation files.	Retrospective coverage spanning 2000–2024.	Structural mapping of the program’s evolution; institutional timeline reconstruction; formulation of initial Theme–CMO pairs
Semistructured Interviews	n = 14 key stakeholders; executive and municipal leaders, case managers, senior social workers, and head nurses, field-based professional caregivers and home-care nurses, informal family caregivers and older adults with mild cognitive decline.	Conducted February–June 2025; mean duration: 90 min.	In-depth exploration of stakeholder reasoning; testing, refining, and validating initial Theme–CMO pairs; uncovering latent motivational mechanisms.
Participant Observation	N = 11 observations; direct participant observations of care visits, multidisciplinary team meetings, community events.	Ongoing throughout 2025; captured via structured field notes.; mean duration: 60 min.	Capturing situated interactions, implicit behavioral patterns, informal institutional routines, and the lived reality of mechanism activation.

**Table 2:** The Five Analytical Domains Interlinking Aging in Place (AiP), Healthy Cognitive Aging (HCA), and Integrated Care (IC). Comprehensive matrix presenting the theoretical definitions of each domain, their specific pathways of interaction regarding cognitive reserve and residential stability, and the operational criteria used for project screening and qualitative analysis.

Analytical Domain	Theoretical Definition (Relation to AiP and HCA)	Operationalization for Screening and Analysis
1. Environmental Stability and Continuity of Care	Predictable and stable micro-environments reduce adaptive stress, reinforcing spatial orientation, memory routines, and psychological safety in cognitively vulnerable seniors.	Tracking the presence of stable personnel assigned to clients, fixed visitation schedules, and long-term organizational durability without disruptive operational modifications.
2. Coordination and integration across health and social care	Fragmented care delivery systems shift the organizational burden onto the older adult or informal caregivers, escalating stress and inducing cognitive overload.	Assessing formal and informal linkages between healthcare and social services, common intake/referral mechanisms, and multidisciplinary communication channels.
3. Social embeddedness and relational support	Regular, meaningful social interactions within a familiar community function as emotional and cognitive anchors, mitigating isolation and slowing cognitive decline.	Examining the structural involvement of informal caregivers, neighbourly support networks, community volunteers, and user participation in local village activities.
4. User autonomy and participation	Autonomy is conceptualized not as a purely atomistic trait, but as a relational achievement that can be scaffolded and preserved even amid cognitive impairment.	Measuring user involvement in individual care planning, respect for subjective pacing/choices in daily routines, and adaptive communication techniques utilized by staff.
5. Cognitive load, stress, and orientation	Complex system navigation drains exigent cognitive reserves and executive capacity; simplifying these pathways optimizes functional independence.	Identifying structural interventions that streamline processes (e.g., single point of entry), provide administrative proxy support, and deliver clear information formats.

**Table 3:** Taxonomic Classification and Relevance Categorisation of Regional Program Projects (2000–2024). Systematic screening matrix of all 43 community-based initiatives developed within the microregion, evaluated for their thematic relevance to Aging in Place (AiP) and Healthy Cognitive Aging (HCA) (Categories A through D) with accompanying institutional justifications for the purposeful selection of the Category D subset.

No.	Title	Relevance Category	Justification for Categorization
1	Establishment of Post-Acute and Long-Term Inpatient Care Services	B	Focuses on institutional, hospital-based bed capacity. While crucial for medical stabilization, it does not directly support aging in place.
2	Development of In-House Specialist Outpatient Clinics	C	Enhances regional healthcare access and reduces long-distance travel, but lacks direct cross-sectoral integration or home-based targeting.
3	Development of Leased Specialist Outpatient Clinics	C	Similar to owned clinics; improves local medical infrastructure for outpatients but operates in isolation from social care domains.
4	On-Site Diagnostic and Clinical Support Services (LAB, X-ray, Ultrasound)	B	Highly technical and diagnostic hospital infrastructure with low direct relevance to behavioral or cognitive aging mechanisms in the community.
5	Provision of Regional Primary Emergency Medical Services	B	Secures acute after-hours medical coverage; provides regional systemic reassurance but does not constitute a continuous care plan for AiP.
6	Provision of Regional Ambulance and Emergency Response Services	B	Urgent care system; essential for physical safety during crises but does not shape daily psychosocial structures or cognitive preservation routines.
7	Facility Renovation A (Inpatient Healthcare Unit)	B	Structural/capital expenditure aimed strictly at institutional inpatient environments within a hospital setting.
8	Facility Renovation B (Inpatient Healthcare Unit)	B	Capital renovation of long-term medical wards; improves institutional quality of care but does not prevent institutionalization.
9	Facility Renovation C (Specialist Clinics, Pharmacy, Accessible Housing)	C	Bridges medical access with physical accessibility features, representing moderate environmental adaptation but segmented service delivery.
10	Facility Renovation D (Long-Term Residential Social Care/Nursing Home in Town Z)	D	Direct infrastructural modification of a community-based residential hub specifically tailored for adults experiencing cognitive decline.
11	Social Work Services Integrated into Post-Acute/Long-Term Inpatient Care	D	Integrates social care practitioners into medical wards, directly reducing organizational workload during complex discharge transitions.
12	Supported Social Housing Linked to Post-Acute/Long-Term Care	D	Pairs secure, accessible housing with ongoing clinical oversight, directly enabling vulnerable individuals to maintain community tenure.
13	Spiritual Care Services (Chapel, Clergy, Chaplaincy)	B	Addresses psycho-spiritual well-being, but operates almost exclusively within the boundaries of the inpatient medical facility.
14	Palliative Care within Post-Acute/Long-Term Inpatient Services	B	Specialized clinical end-of-life care on dedicated hospital beds; although exhibiting high relational continuity, it inherently operates within an institutional environment.
15	Targeted Care Interventions (Goal-Oriented Care Framework)	C	System-wide introduction of goal-oriented care pathways; enhances user participation but was primarily piloted within institutional segments.
16	Clinical Care Provision in Long-Term Residential Social Care (Nursing Home D)	D	Embeds skilled nursing care directly into a social facility, preventing disruptive hospitalizations and consequent acute disorientation.
17	Regional Social Rehabilitation and Assistive Device Loan Service	D	Provides an immediate, cost-free transitional safety net by supplying physical compensatory devices during the critical shift from hospital to home. It absorbs systemic friction, eliminates procurement stress, and prevents care environment collapse prior to official health insurance approvals.
18	Transition of Social Services to the Regulatory Framework under Act No. 108/2006	A	Purely administrative, structural, and regulatory adjustment to national legislation with no direct behavioral impact on clients.
19	Home Care and Personal Assistance Services	D	A core pillar of AiP. Implements regular home care visits to stabilize daily domestic routines and preserve familiar environments.
20	Social Activation and Community Engagement Services	D	Directly targets healthy cognitive aging through structured memory exercises, cognitive stimulation, and continuous social integration.
21	Personal Assistance (Individual Support Services)	D	Provides high-intensity individual support tailored to the client’s home life, maximizing user autonomy and active participation.
22	Home-Based Hospice Care	D	Facilitates end-of-life care within the domestic setting, minimizing acute care transfers and protecting relational stability.
23	Assistive Technology Showroom and Advisory Centre	C	Operates as a discrete informational and consultative infrastructure allowing clients and caregivers to test adaptive technologies. While it effectively reduces knowledge barriers and systemic friction, it does not directly deliver home-based care or operationally modify the domestic setting.
24	Volunteer Programs and Community Volunteering	D	Mobilizes informal community networks, providing emotional anchoring and preventing isolation among solitary older adults.
25	Animal-Assisted Therapy and Pet Programs in Healthcare Settings	C	Goal-directed animal therapy; increases positive emotional/cognitive responses but is deployed predominantly in a hospital setting.
26	Home Health Care Services	D	Deploys skilled community nurses to execute clinical tasks at home, removing the stress of outpatient travel and stabilizing routines.
27	Long-Term Residential Social Care (Nursing Home in Town Y)	C	Residential social care facility located outside the primary microregion; provides safety but separates the individual from local roots.
28	Respite Care Services	D	Mitigates burnout among informal family caregivers, preserving the informal support structure essential for long-term domestic AiP.
29	Specialized Residential Care Facility I (Dementia-Specific Care)	D	A residential service custom-designed for adults with advanced dementia, focusing extensively on environmental orientation cues.
30	Supported Apartments within Facility C	D	Implements sheltered community living where clients reside in individual apartments but have immediate access to integrated care teams.
31	Staff Accommodation within Facility C	C	Promotes organizational stability by providing staff housing, which indirectly supports continuity of care but lacks direct client impact.
32	Community Garden in Town Z	D	An adapted outdoor environment supporting safe physical activity, peer-to-peer socialization, and informal cognitive stimulation.
33	Local Care Cottage A (Small-Scale Community Care Dwelling)	D	Decentralised, low-capacity housing units enabling ageing in place with mobile care, preserving local identity and neighbourly ties in village A.
34	Local Care Cottage B (Small-Scale Community Care Dwelling)	D	Decentralised, low-capacity housing units enabling ageing in place with mobile care, preserving local identity and neighbourly ties in village B.
35	Local Care Cottage C (Small-Scale Community Care Dwelling)	D	Decentralised, low-capacity housing units enabling ageing in place with mobile care, preserving local identity and neighbourly ties in village C.
36	Local Care Cottage D (Small-Scale Community Care Dwelling)	D	Decentralised, low-capacity housing units enabling ageing in place with mobile care, preserving local identity and neighbourly ties in village D.
37	Local Care Cottage E (Small-Scale Community Care Dwelling)	D	Decentralised, low-capacity housing units enabling ageing in place with mobile care, preserving local identity and neighbourly ties in village E.
38	Local Care Cottage in Town Z	D	Small-capacity urban sheltered housing unit promoting localized community integration and flexible personal pacing.
39	Operational Base for Home Care Services in Village G	D	A localized operational base that minimizes travel times for field nurses, ensuring responsive and highly reliable home care.
40	Specialized Residential Care Facility II (Alternative Site)	D	A satellite facility specialized in advanced dementia care, featuring customized sensory and architectural orientation elements.
41	Comprehensive Reconstruction of Facility B (Inpatient Healthcare Unit)	B	Large-scale structural modernizing of long-term medical hospital wards; improves institutional parameters but remains institutional.
42	Provision of Nursing Staff in Residential Social Care Facilities	D	Personnel integration deploying professional nurses into social care homes, mitigating the stress of frequent diagnostic transfers.
43	Provision of Medical Staff (Physicians) in Residential Social Care Facilities	D	Guarantees regular, on-site medical rounds within residential care homes, fostering strong relational continuity and an enhanced sense of safety.

**Table 4:** Methodological Exclusion and Inclusion Criteria for Project Categorisation. Operational matrix defining the structural, administrative, and behavioral boundaries used to classify the 43 regional projects into Relevance Categories A, B, C, and D based on their proximal capacity to modify environmental cues or activate individual pathways to Aging in Place (AiP) and Healthy Cognitive Aging (HCA).

**Category A: No/Minimal Relevance**
○ **Criterion:** Projects of a purely legislative, formal-transformational, or macro-administrative character. These interventions operate at the structural level of the system and do not interact with the micro-environment or the daily behaviors of the older adult. They entail no direct modification of care delivery in situ, do not alter physical or social cues in the client’s immediate surroundings, and lack any measurable behavioral impact on the target population. From the perspective of the AiP-HCA synergy, these projects are irrelevant as they fail to influence individual executive capacity or cognitive reserve pathways.
**Category B: Low/Indirect Relevance (Institutional Healthcare Infrastructure)**
○ **Criterion:** Projects focused on acute, urgent, or generic long-term inpatient care within hospital environments, including its technical and logistical infrastructure. Although these interventions are critical for the macro-systemic stabilization of regional healthcare and the maintenance of baseline physiological parameters, they inherently remove the individual from their natural ecological context. By definition, such projects fall outside the scope of community tenure (AiP), as they involve institutionalization rather than continued residence in the community. Within the classification framework applied in this study, these projects are categorized as not directly targeting the psychosocial mechanisms of cognitive aging, not leveraging procedural memory through familiar environmental cues, and not addressing the stabilization of informal caregiving networks. These are categorization criteria rather than empirically demonstrated limitations, as Category B projects were not qualitatively analysed in the present study.
**Category C: Medium Relevance (Specialized Outpatient or Segmented Services)**
○ **Criterion:** Projects developing discrete outpatient specialist services, isolated infrastructural modifications, or services located outside the primary microregion. From a behavioral standpoint, these projects reduce specific operational friction points and logistical fatigue (e.g., eliminating taxing travel for medical consultations, which secondarily preserves an individual’s cognitive reserve). However, they lack deep, continuous cross-sectoral integration (the synchronization of health and social care axes) and do not systematically reconfigure the daily choice architecture within the home or community. Consequently, their impact on the behavioral routines that couple AiP and HCA remains episodic and fragmented rather than continuous.
**Category D: High Relevance (Core Direct AiP/HCA Interventions)**
○ **Criterion:** Projects directly implementing field-based, domestic, or small-scale decentralized community services that functionally and operationally integrate the health and social care sectors. These interventions represent the operational core of the conceptual AiP-HCA nexus. From a behavioral perspective, they actively function as “cognitive scaffolding”; they systematically absorb the older adult’s executive and organizational burden (e.g., via single-point-of-entry mechanisms), eliminate navigation-induced stress within complex systems, and insulate the client from cognitive overload. By ensuring staff continuity and predictable care schedules, these projects reinforce environmental predictability, allowing individuals to rely on procedural memory and maintain deeply ingrained behavioral routines. Through the scaffolding of relational autonomy and the mobilization of localized social anchors (neighborly networks, volunteers), these interventions explicitly link prolonged independent domestic tenure (AiP) with the environmental conditions necessary for sustaining cognitive functioning in the community (HCA). This subset constitutes the analytical core of the realist evaluation.

**Table 5:** Heuristic Matrix of Developed Context–Mechanism–Outcome (CMO) Configurations. Detailed systematic register of the 24 qualitative configurations derived from the iterative triangulation of documentary records, semi-structured stakeholder interviews (N = 14), and participant field observations (N = 11), structured across the core analytical domains.

No.	Analytical Domain	Context (C)	Mechanism (M) (Role of IC as a Conditional Moderator)	Outcome (O)	Evidence/Audit Trail
**1**	**1. Environmental Stability and Continuity of Care**	Rural microregion with long-term personnel stability in domiciliary care services.	Staff familiarity triggers the user’s procedural memory, minimizing anxiety and stabilizing routines.	Behavioral stabilization and maintenance of independent domestic tenure (AiP/conditions relevant to HCA).	**Doc:** Care Provider Manual. **Int:** OA1 (Older Adult) on caregiver “J” **Obs:** Recognition during home visit.
**2**	**1. Environmental Stability and Continuity of Care**	Fixed, rigid time schedules for home care visits embedded within a long-established Municipal Program.	Temporal structuring compensates for impaired time orientation, reinforcing predictability and ontological security.	Heightened sense of safety and elimination of anticipatory stress in daily life (AiP).	**Doc:** Care Plan (fixed times). **Int:** PC2 (Professional Caregiver) on window watching. **Obs:** Medication ready 5 min before visit.
**3**	**1. Environmental Stability and Continuity of Care**	Availability of decentralized, community-based care homes (Local Care Cottages) in affiliated municipalities.	Geographic anchoring preserves local spatial and social ties, eliminating the need for comprehensive reorientation.	Conditions supporting the reduction in relocation stress and prolongation of living in the familiar community (AiP/conditions relevant to HCA).	**Doc:** Strategic plan (Cottages). **Int:** OA2 (Older Adult) on village roots. **Obs:** Proximity of cottage to former home.
**4**	**1. Environmental Stability and Continuity of Care**	Continuous, on-site nursing care directly within a localized social facility (Facility D).	Clinical continuity enables proactive monitoring by familiar staff, fostering relational trust and security.	Reduced emergency hospitalizations and stabilization of health fluctuations (AiP/conditions relevant to HCA).	**Doc:** Facility D nursing protocol. **Int:** HN1 (Head Nurse) on monitoring. **Obs:** Daily clinical rounds.
**5**	**1. Environmental Stability and Continuity of Care**	25-year institutional stability of the voluntary municipal association ensuring sustainable funding.	Institutional permanence cultivates systemic trust, absorbing existential anxiety and releasing executive capacity.	Facilitation of proactive long-term planning and reduction in existential stress (AiP/conditions relevant to HCA).	**Doc:** Founding Charter. **Int:** ML1 (Municipal Leader) on systemic trust. **Obs:** Budget consensus at board.
**6**	**1. Environmental Stability and Continuity of Care**	Concurrent co-provision of standard home care and specialized domestic hospice services.	Care convergence prevents domestic chaos and service overlap through joint intervention planning and coordination.	Preservation of environmental tranquility and maximum stability during end-of-life (AiP/conditions relevant to HCA).	**Doc:** Integrated hospice protocols. **Int:** PC1 (Professional Caregiver) on joint visits. **Obs:** Coordination in domestic setting.
**7**	**2. Coordination and integration across health and social care**	Integrated transition pathways and multidisciplinary case conferences within the microregion.	Transition management through seamless information sharing eliminates communication gaps and navigational friction.	Flexible adaptation of care plans without disrupting support continuity during transitions (AiP).	**Doc:** Multidisciplinary board minutes. **Int:** CM1 (Case Manager) on transition care. **Obs:** Discharge planning meeting.
**8**	**2. Coordination and integration across health and social care**	Discharge from post-acute inpatient healthcare back into the domestic environment.	Early social work engagement (Project 11) reduces family logistical stress and prepares the home environment.	Seamless hospital-to-home transition, minimizing risk of immediate care failure (AiP).	**Doc:** Project 11 social work files. **Int:** CM1 (Case Manager) on early engagement. **Obs:** Home modification assessment.
**9**	**2. Coordination and integration across health and social care**	Convergence of specialized outpatient clinics and home healthcare services under single administration.	Shared information systems relieve the family from acting as intermediaries, enhancing medication safety and clinical oversight.	Enhanced medication adherence and reduction in psycho-social burden on caregivers (AiP/conditions relevant to HCA).	**Doc:** Shared record protocol. **Int:** N1 (Home-care Nurse) on clinician comms. **Obs:** Home medication check.
**10**	**2. Coordination and integration across health and social care**	Centralized referral system and shared indication pathways governed by the municipal association.	Unified navigation removes provider competition, increasing the sense of systemic predictability and safety.	Comprehensive coverage of client needs without fragmentation, stabilizing community tenure (AiP).	**Doc:** Centralized referral guidelines. **Int:** ML2 (Municipal Leader) on entry pathways. **Obs:** Shared intake procedure.
**11**	**3. Social embeddedness and relational support**	Geographically dispersed households in a microregion with high demographic aging.	Field mobilization and volunteer networks establish social anchors, activating community solidarity and affective ties.	Contextual conditions facilitating social connectedness and loneliness as risk factors for cognitive decline (conditions relevant to HCA).	**Doc:** Volunteer methodology. **Int:** SW1, Social Worker (+Volunteer V1) on Mr. White. **Obs:** Interaction on village porch.
**12**	**3. Social embeddedness and relational support**	Accessible community garden and social activation services in the microregion’s hub (Town Z).	Civic activation through shared meaningful occupations stimulates neuroplasticity and peer bonding.	Enhanced socially and cognitively engaging activities supporting cognitive reserve (AiP/conditions relevant to HCA).	**Doc:** Community garden project file. **Int:** OA2 (Older Adult) on feeling useful. **Obs:** Social activation sessions.
**13**	**3. Social embeddedness and relational support**	Small, tight-knit village context where residents share lifelong acquaintances.	Natural surveillance by the neighborhood functions as an informal early warning network for cognitive or physical changes.	Early detection of emergent cognitive changes (e.g., wandering) and rapid professional response (AiP/conditions relevant to HCA).	**Doc:** Village solidarity report. **Int:** IC1 (Informal Caregiver) on surveillance. **Obs:** Informal peer-to-peer check.
**14**	**4. User autonomy and participation**	Collaborative formulation of individualized care plans (Project 21) focused on preserved capabilities.	Resource-oriented planning scaffolds relational autonomy, reinforcing the user’s internal locus of control and agency.	Prevention of learned helplessness and support for everyday executive planning (AiP/conditions relevant to HCA).	**Doc:** Skill-based assessment form. **Int:** SW2 (Social Worker) on scaffolding walk. **Obs:** Supporting self-dressing.
**15**	**4. User autonomy and participation**	Older adult with cognitive impairment who insists on maintaining unconventional personal habits.	Validating communication and flexible staff response respect client individuality, reducing interpersonal conflict.	Mitigation of resistance to care and strengthening of the therapeutic partnership (AiP).	**Doc:** Training module (validating). **Int:** PC2 (Professional Caregiver) on routine respect. **Obs:** Non-confrontational interaction.
**16**	**4. User autonomy and participation**	Progressive decline in physical capacity compounded by cognitive frailty in an older adult living alone.	Environmental mastery via assistive technologies (Project 17) enhances self-efficacy and reduces fear of functional failure.	Prolongation of independence in ADLs and reduction in care dependency (AiP).	**Doc:** Device loan records. **Int:** PC1 (Professional Caregiver) on equipment use. **Obs:** Practice with walker at home.
**17**	**4. User autonomy and participation**	Co-design and evaluation of community-based activation programs within the microregion.	Participatory inclusion in governance validates lived experience, enhancing the user’s sense of social utility and agency.	Reinforcement of narrative identity and resilience against aging-related cognitive withdrawal (conditions relevant to HCA).	**Doc:** User feedback board minutes. **Int:** OA2 (Older Adult) on agency. **Obs:** Co-design workshop participation.
**18**	**5. Cognitive load, stress, and orientation**	Highly complex, fragmented health/social benefits and eligibility criteria in the Czech Republic.	Single point of entry acts as a system-wide sponge, fully absorbing the logistical and organizational burden.	Elimination of navigational stress and prevention of executive overload in system access (conditions relevant to HCA/AiP).	**Doc:** ‘One Door’ info leaflet. **Int:** IC2 (Informal Caregiver) on bureaucracy relief. **Obs:** Unified coordination at intake.
**19**	**5. Cognitive load, stress, and orientation**	Daily executive management of household operations under early-stage dementia.	Practical proxy support (e.g., social rehabilitation) scaffolds working memory by assisting with bills and grocery logistics.	Minimization of chronic distress and buffering against catastrophic executive failures in daily life (AiP/conditions relevant to HCA).	**Doc:** Social rehabilitation file. **Int:** SW2 (Social Worker) on financial scaffolding. **Obs:** In-home administrative help.
**20**	**5. Cognitive load, stress, and orientation**	Navigation of an older adult within barrier-free housing or a localized community hub.	Spatial simplification via intuitive visual cues and architectural clarity reduces environmental stress and ambiguity.	Facilitation of orientation and enhancement of perceived safety in neighborhood mobility (AiP/conditions relevant to HCA).	**Doc:** Architectural cues guidelines. **Int:** ML1 (Municipal Leader) on visual cues. **Obs:** Client navigating using cues.
**21**	**5. Cognitive load, stress, and orientation**	Regular delivery of structured memory training and cognitive stimulation within activation services.	Cognitive scaffolding through micro-tasks calibrated to executive capacity stimulates residual cognitive reserve.	Enhanced mastery of everyday tasks via compensatory strategies and reduced frustration (conditions relevant to HCA).	**Doc:** Social activation logs. **Int:** PC2 (Professional Caregiver) on micro-tasks. **Obs:** Structured cognitive training.
**22**	**5. Cognitive load, stress, and orientation**	Acute domestic crises (e.g., sudden deterioration or absence of primary caregiver) in a remote village.	Crisis de-escalation via 24/7 hotline and rapid mobile teams provides an immediate emotional and logistical safety net.	Mitigation of acute panic and prevention of unnecessary emergency institutionalization during crisis (AiP/conditions relevant to HCA).	**Doc:** 24/7 hotline operational log. **Int:** IC2 (Informal Caregiver) on crisis response. **Obs:** Mobile team intervention.
**23**	**5. Cognitive load, stress, and orientation**	High psychosocial and emotional burden on family members caring for relatives with dementia.	Caregiver protection through respite services and counseling alleviates burnout, stabilizing the informal care structure.	Delaying institutionalization through prolonged stabilization of the home caregiving environment (AiP/conditions relevant to HCA).	**Doc:** Respite care evaluation. **Int:** IC1 (Informal Caregiver) on burnout relief. **Obs:** Caregiver counseling session.
**24**	**5. Cognitive load, stress, and orientation**	Implementation of targeted volunteer initiatives, including animal-assisted interventions.	Tactile activation and non-verbal communication elicit positive emotional responses, improving behavioral adaptability.	Reduction in manifestations of agitation and apathy in daily domestic cohabitation (conditions relevant to HCA).	**Doc:** Animal therapy report. **Int:** SW1, Social Worker (+Volunteer V1) on non-verbal cues. **Obs:** Interaction with therapy dog.

**Table 6:** Conceptual Consolidation of Empirical CMO Configurations into Core Support Pillars. This integrative matrix synthesizes how specific operational context–mechanism dynamics cluster into seven primary pillars of support, mapping the transition from empirical data to theoretical abstraction regarding conditions relevant to aging in place (AiP) and healthy cognitive aging (HCA).

Theoretical Pillar	Core CMO	Supporting/Absorbed CMOs	Synthesized Generative Mechanism (Role of IC as a Conditional Moderator)	Representative Empirical Evidence (Illustrative Case)
**1. Institutional & Governance Bedrock**	**CMO 5**	None	Long-term institutional stability and solidarity-based funding generate systemic trust, allowing families to engage in proactive long-term planning and releasing executive capacity to manage daily tasks.	**Doc:** Strategic Plan declaring long-term commitment. **Int:** ML2 (Municipal Leader) on deep systemic trust. **Obs:** Board meeting consensus on future budget.
**2. Relational Continuity & Memory Scaffolding**	**CMO 1**	**CMO 4, CMO 6**	High staff stability leverages procedural memory and interpersonal familiarity, minimizing acute anxiety and stabilizing domestic routines for cognitively vulnerable adults.	**Doc:** Care Provider Manual. **Int:** OA1 (Older Adult) on caregiver “J” **Obs:** Recognition during home visit.
**3. Environmental & Temporal Predictability**	**CMO 2**	**CMO 3** *(Reconciled)*	Strict temporal scheduling and geographic anchoring compensate for disorientation in time and space, reinforcing a sense of ontological security within the micro-environment.	**Doc:** Care Plan (fixed times). **Int:** PC2 (Professional Caregiver) on window watching. **Obs:** Medication ready 5 min before visit.
**4. Cross-Sectoral Coordination**	**CMO 10**	**CMO 7, CMO 8, CMO 9**	The elimination of administrative silos and multi-professional alignment prevents service fragmentation during transitions, establishing conducive conditions for safe community tenure.	**Doc:** Inter-sectoral Agreement on unified intake. **Int:** CM1 (Case Manager) on coordinating health and social needs. **Obs:** Multidisciplinary discharge case conference.
**5. Socio-Relational Embeddedness**	**CMO 11**	**CMO 12, CMO 13**	Mobilization of field staff and volunteers creates a social safety net that functions as a buffer against chronic loneliness, a theorised risk factor for accelerated cognitive decline.	**Doc:** Volunteer Methodology for social partnership. SW1, Social Worker (+Volunteer V1) on Mr. White. **Obs:** Senior/volunteer bonding in community garden.
**6. Supported Autonomy and Agency**	**CMO 14**	**CMO 15, CMO 16, CMO 17**	Shifting from paternalistic care to goal-directed planning protects the user’s sense of agency and motivates the maintenance of residual executive capacities.	**Doc:** Assessment Form mapping preserved skills. **Int:** SW2 (Social Worker) on scaffolding a client’s walk to a shop. **Obs:** Caregiver waiting for client to fasten buttons.
**7. Reduction In Cognitive & Navigational Burden**	**CMO 18**	**CMO 19, CMO 20, CMO 21, CMO 22, CMO 23, CMO 24**	A centralized point of entry and spatial simplification (CMO 20) absorb logistical complexity, shielding the individual from executive overload and care environment collapse.	**Doc:** Leaflet ‘One Door for All Needs’. **Int:** IC2 (Informal Caregiver) on power of attorney for bureaucracy. **Obs:** Social worker organizing bill folders for senior.

## Data Availability

The data presented in this study are available on request from the corresponding author due to privacy and ethical restrictions, as they contain sensitive qualitative information that could compromise participant anonymity.

## Abbreviations

The following abbreviations are used in this manuscript:

AiP	Aging in Place
HCA	Healthy Cognitive Aging
IC	Integrated care
CMO	Context-Mechanism-Outcome
ADL	Activities of Daily Living
STAC	Scaffolding theory of aging and cognition
MMSE	Mini-Mental State Examination
MoCA	Montreal Cognitive Assessment
